# Novel nonsteroidal topical therapies for pediatric atopic dermatitis: a systematic review and network meta-analysis of randomized controlled trials

**DOI:** 10.3389/fimmu.2026.1906218

**Published:** 2026-07-22

**Authors:** Yuheng Yang, Dan Zhao, Yicheng Wang, Qin Yang

**Affiliations:** 1 Drug and Equipment Section, Chengdu Children Special Hospital, Chengdu, Sichuan, China; 2 Department of Physiology, West China School of Basic Medical Sciences and Forensic Medicine, Sichuan University, Chengdu, Sichuan, China; 3 Chengdu MKZ Biotechnology Co., Ltd, Chengdu, Sichuan, China; 4 Department of Reproductive Health and Infertility, Chengdu Women’s and Children’s Central Hospital, School of Medicine, University of Electronic Science and Technology of China, Chengdu, Sichuan, China; 5 Department of Dermatovenereology, West China Longquan Hospital Sichuan University/The First People's Hospital of Longquanyi District Chengdu, Chengdu, Sichuan, China

**Keywords:** atopic dermatitis, network meta-analysis, nonsteroidal topical therapy, pediatric dermatology, steroid-sparing treatment

## Abstract

**Background:**

Novel nonsteroidal topical therapies are increasingly used for pediatric atopic dermatitis (AD), but their comparative efficacy and safety remain unclear because direct active-comparator evidence is limited.

**Methods:**

We conducted a systematic review and frequentist network meta-analysis (NMA) of randomized controlled trials (RCTs) evaluating novel nonsteroidal topical therapies in children with AD. The study was registered in PROSPERO (CRD420261400624). Efficacy outcomes included Investigator’s Global Assessment treatment success (IGA-TS), Eczema Area and Severity Index 75 response (EASI-75), and EASI-90. Safety outcomes included treatment-emergent adverse events (TEAEs) and treatment-related adverse events (TRAEs). Treatment nodes were defined by drug, concentration, and dosing frequency. Pairwise vehicle-controlled meta-analyses, sensitivity analysis, and subgroup analyses by treatment duration and age were also performed.

**Results:**

Twelve articles reporting 15 RCTs involving 3,994 pediatric patients were included. The network was largely centered on vehicle, with only one active-comparator trial. Most active regimens improved IGA-TS versus vehicle, except tapinarof 0.5% once daily. For EASI-75, five of ten evaluated active regimens showed significant benefit, while all six regimens evaluated for EASI-90 were superior to vehicle. Delgocitinib 0.5% twice daily ranked highest for IGA-TS and EASI-75, whereas difamilast 1% twice daily ranked highest for EASI-90. No consistent increase in TEAEs or TRAEs was observed. Pairwise meta-analyses and sensitivity analysis were generally consistent with the primary findings. Subgroup analyses suggested timepoint- and age-related differences, but stratified evidence was limited.

**Conclusion:**

Novel nonsteroidal topical therapies provide effective short-term steroid-sparing options for pediatric AD without a clear overall safety penalty. Further head-to-head, long-term, and age-stratified trials are needed.

**Systematic review registration:**

https://www.crd.york.ac.uk/PROSPERO/view/, identifier CRD420261400624.

## 1 Introduction

Atopic dermatitis (AD) is one of the most common chronic inflammatory skin diseases in childhood, with a global point prevalence of 11.1% among children and adolescents (1). Although AD is not typically life-threatening, its hallmark features, including persistent pruritus, recurrent eczematous lesions, sleep disturbance, and visible skin inflammation, can place a substantial physical, psychological, and socioeconomic burden on affected children and their families (2). Over the past decade, AD has increasingly been understood not as a nonspecific inflammatory dermatosis, but as a heterogeneous disease shaped by the interplay between epidermal barrier dysfunction, type 2 immune activation, neuroimmune itch signaling, and cutaneous microbial dysbiosis (3, 4). This more refined understanding of disease biology has opened the door to mechanism-directed therapies, ranging from systemic biologics and small molecules to novel nonsteroidal topical agents (5, 6).

Topical therapy remains the foundation of AD management in children, particularly for mild-to-moderate disease and localized flares. Conventional care has long relied on regular emollient use to restore barrier function and topical corticosteroids (TCSs) to suppress active inflammation (5, 7). Although TCSs are effective, inexpensive, and familiar to clinicians, their repeated use in children is often complicated by concerns about skin atrophy, use on thin or sensitive skin sites, and caregiver anxiety about corticosteroid-related adverse effects (8, 9). Topical calcineurin inhibitors (TCIs), including tacrolimus and pimecrolimus, added an important steroid-sparing option, particularly for the face, eyelids, neck, flexures, and other areas where prolonged corticosteroid exposure may be undesirable. However, TCIs are not a complete solution: application-site burning or stinging, variable tolerability, and persistent concerns related to their boxed warning may limit their acceptance in routine pediatric practice (7). More recently, the topical treatment landscape has expanded beyond corticosteroids and TCIs with the development of novel nonsteroidal agents, including phosphodiesterase-4 (PDE4) inhibitors, topical Janus kinase (JAK) inhibitors, and aryl hydrocarbon receptor (AhR) agonists (6). These agents reflect a shift from broad anti-inflammatory suppression toward more mechanism-directed topical intervention, with the practical appeal of local delivery and reduced need for systemic exposure.

PDE4 inhibitors, such as crisaborole and roflumilast, increase intracellular cyclic adenosine monophosphate signaling and dampen the production of proinflammatory cytokines, offering a topical anti-inflammatory strategy with broad applicability in mild-to-moderate disease (10, 11). Topical JAK inhibitors, represented by ruxolitinib cream, target JAK-STAT signaling downstream of key AD-related cytokines and may provide more direct suppression of inflammatory and itch-related pathways (12). In contrast, AhR agonists, such as tapinarof, act through a pathway linked to epidermal differentiation, barrier homeostasis, oxidative stress regulation, and immune modulation, making this class conceptually distinct from conventional anti-inflammatory topical agents (13). This expanding therapeutic landscape gives clinicians more options, but it also creates a practical problem. Most pediatric trials were designed to compare a single active treatment with its vehicle, and direct head-to-head comparisons among these novel agents remain scarce. As a result, the relative efficacy, tolerability, and benefit-risk balance of these treatments in children remain difficult to judge. We therefore conducted a systematic review and network meta-analysis (NMA) of randomized controlled trials (RCTs) to compare the efficacy and safety of novel nonsteroidal topical therapies for pediatric AD.

## 2 Methods

This systematic review and NMA was conducted and reported in accordance with the Preferred Reporting Items for Systematic Reviews and Meta-Analyses (PRISMA) extension statement for NMA (14), and the completed reporting checklist is provided in **Supplementary Table 1**. The study protocol was prospectively registered in the International Prospective Register of Systematic Reviews (PROSPERO; registration number: CRD420261400624). No ethics approval was required because this study was based exclusively on published aggregate data.

### 2.1 Eligibility criteria and search strategy

The eligibility criteria were defined according to the population, intervention, comparator, outcome, and study design (PICOS) framework. Eligible studies were RCTs that enrolled pediatric patients with AD and evaluated novel nonsteroidal topical therapies, including topical PDE4 inhibitors, JAK inhibitors, and AhR agonists. Trials were eligible if they compared an active treatment with vehicle, placebo, or another active topical comparator. Studies enrolling mixed adult and pediatric populations were considered only when pediatric data could be extracted separately or when the pediatric subgroup was clearly reported.

The primary efficacy outcomes of interest were Investigator’s Global Assessment treatment success (IGA-TS), Eczema Area and Severity Index 75 response (EASI-75), and Eczema Area and Severity Index 90 response (EASI-90). Safety outcomes included treatment-emergent adverse events (TEAEs) and treatment-related adverse events (TRAEs). Studies were excluded if they were nonrandomized studies, observational studies, case reports, reviews, editorials, animal or *in vitro* studies, studies evaluating systemic therapy alone, phototherapy, traditional TCSs, or TCIs as the main intervention, or studies without extractable pediatric efficacy or safety data.

A comprehensive literature search was performed in PubMed, Embase, Cochrane Library, and Web of Science from database inception to May 31, 2026. Search terms combined controlled vocabulary and free-text terms related to atopic dermatitis, pediatric populations, topical therapy, and specific drug classes or agents, including PDE4 inhibitors, JAK inhibitors, AhR agonists, crisaborole, roflumilast, ruxolitinib, and tapinarof. The detailed search strategy for each database is presented in **Supplementary Table 2**. Reference lists of relevant reviews and included studies were also screened to identify additional eligible records.

After duplicate records were removed, two reviewers (YH Yang and D Zhao) independently screened titles and abstracts, followed by full-text assessment of potentially eligible articles. Disagreements were resolved through discussion or consultation with a third reviewer (YC Wang).

### 2.2 Data extraction

Two reviewers (YH Yang and D Zhao) independently extracted data from each eligible study using a prespecified standardized data extraction form. Extracted information included the first author, publication year, trial name, country or region, study phase, blinding status, comparator type, sample size, pediatric age subgroup, baseline disease severity, treatment class, drug name, dose or concentration, dosing frequency, treatment duration, assessment time point, and numbers of participants included in efficacy and safety analyses. For each treatment arm, we extracted the number of participants with IGA-TS, EASI-75, EASI-90, TEAEs, and TRAEs. IGA-TS was extracted according to the definition used in each trial, generally defined as a score of clear or almost clear with at least a 2-grade improvement from baseline. EASI-75 and EASI-90 responses were defined as at least 75% and 90% reductions, respectively, in EASI score from baseline.

When trials reported multiple time points, data at the end of the randomized treatment period were used for the primary analysis, and data at specific time points were extracted for planned subgroup analyses when available. For studies enrolling both pediatric and adult participants, only pediatric data were extracted when separately reported. When multiple publications reported results from the same trial, data were cross-checked and extracted from the most complete or most recent report to avoid duplication. Any discrepancies in data extraction were resolved by discussion, and unresolved disagreements were adjudicated by a third reviewer (YC Wang).

### 2.3 Risk-of-bias, transitivity, heterogeneity, and inconsistency assessments

The risk of bias of included RCTs was assessed independently by two reviewers (D Zhao and Q Yang) using the version 2 of the Cochrane risk-of-bias tool for randomized trials (RoB 2) (15). Disagreements were resolved through discussion or consultation with a third reviewer (YC Wang).

The transitivity assumption was assessed by comparing key clinical and methodological characteristics across treatment comparisons, including age range, baseline disease severity, treatment duration, outcome assessment time point, control type, blinding status, and study phase. These characteristics were examined to determine whether participants included in different comparisons were sufficiently similar to support valid indirect comparisons across the treatment network. For conventional pairwise meta-analyses, between-study heterogeneity was assessed using Cochran’s Q test and the I^2^ statistic. For NMA, between-study heterogeneity was evaluated using the estimated between-study variance under the random-effects model. Global inconsistency was assessed using a design-by-treatment interaction model when the network structure allowed. Local inconsistency was examined using node-splitting or side-splitting approaches for comparisons with both direct and indirect evidence.

### 2.4 Statistical analysis

Conventional pairwise meta-analyses were first performed for direct comparisons between active treatments and comparators. Random-effects models were used to account for expected clinical and methodological heterogeneity across trials. For each outcome, pooled estimates were generated when at least two studies were available for a given comparison. In addition to individual treatment-level comparisons, pairwise meta-analyses were summarized at the drug-class level and overall level to provide a descriptive overview of the direct evidence for novel nonsteroidal topical therapies.

NMA were then conducted within a frequentist framework using random-effects models. Treatment nodes in the primary NMA were defined according to active drug, concentration, and dosing frequency to preserve clinically relevant differences among topical regimens. Vehicle arms were combined into a common comparator node because vehicle-controlled trials accounted for most of the available evidence and served as the shared reference across the network. The primary NMA used data collected at the end of the randomized treatment period. Prespecified sensitivity and subgroup analyses were conducted to examine the robustness and clinical consistency of the findings.

All outcomes were analyzed as dichotomous variables. Effect estimates were expressed as risk ratios (RRs) with 95% confidence intervals (CIs). For NMA, treatment rankings were estimated using P-scores, with higher P-scores indicating a greater probability of being among the most favorable treatment options. For studies with zero events in one treatment arm, a constant continuity correction of 0.5 was applied to allow estimation of the treatment effect. Confidence in the primary active-versus-vehicle network estimates was assessed using the Confidence in Network Meta-Analysis (CINeMA) framework (16, 17). The assessment covered the primary efficacy and safety outcomes. All statistical analyses were performed using R software, version 4.6.0, with the “meta” and “netmeta” packages.

## 3 Results

### 3.1 Study selection and characteristics

**Figure 1:**
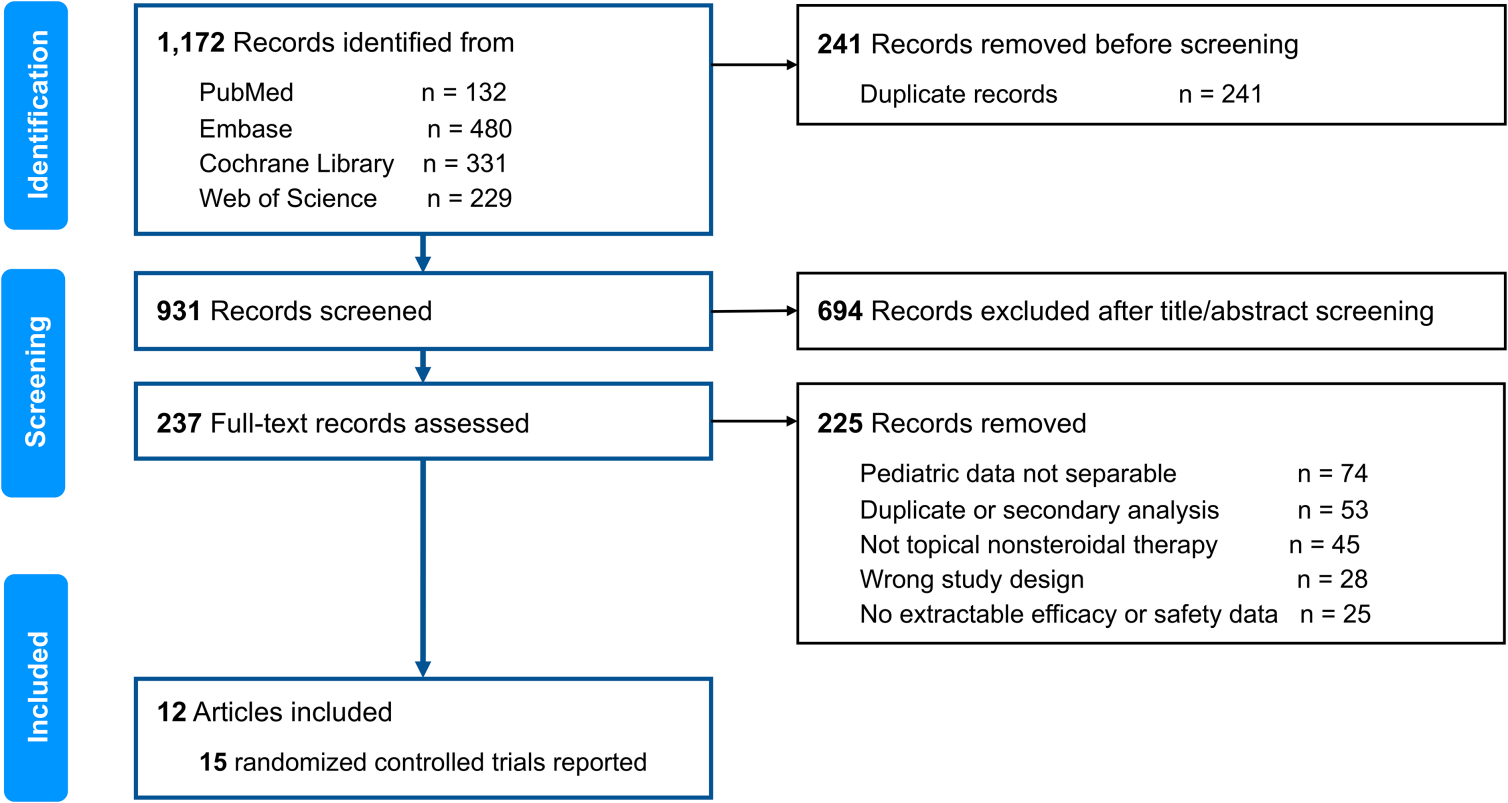
PRISMA flow diagram of study identification, screening, and inclusion.

The study selection process is shown in **Figure 1**. After database searching and manual screening, 1172 records were identified, 241 duplicates were removed, and 694 excluded after screening with title and abstract. 237 full-text articles were assessed for eligibility, of which 225 were excluded for the reasons detailed in **Figure 1**. Finally, 12 articles reporting 15 RCTs were included, comprising 3,994 pediatric patients with AD aged 2 to younger than 18 years (18–29). Overall, 14 trials were randomized, double-blind, vehicle-controlled phase 2 or phase 3 studies and were judged to have a low risk of bias using the RoB 2 tool (**Supplementary Figure 1**).

The main characteristics of the included trials are summarized in **Table 1**. Most enrolled patients had mild-to-moderate AD at baseline, although a small proportion of patients with severe disease were included in selected studies. Seven trials enrolled mixed adult and pediatric populations with extractable pediatric data, whereas eight trials were conducted exclusively in pediatric patients. The included novel nonsteroidal topical therapies covered three mechanistic classes: PDE4 inhibitors, including crisaborole, difamilast, and roflumilast; topical JAK inhibitors, including delgocitinib and ruxolitinib; and an AhR agonist, tapinarof. All included PDE4 inhibitor trials used a 4-week treatment period. Among topical JAK inhibitor trials, ruxolitinib was evaluated over 8 weeks, whereas delgocitinib was evaluated over 4 weeks. The AhR agonist tapinarof was evaluated in 8-week trials.

**Table 1:** Characteristics of the included studies.

Study	Clinical trial identifier	Country or region	Study phase	Study design	Population	Study treatment	Mechanistic class	Treatment duration, weeks	Pediatric sample size	Pediatric age, years (mean or median)	Baseline IGA severity	Moderate IGA at baseline, %	Baseline EASI score (mean)	Baseline affected BSA, % (mean)
Atsuyuki, 2025 (20)	jRCT2031210257	Japan	Phase 2	Double-blind, randomized, vehicle-controlled	Pediatric only	Tapinarof, QD, 0.5% and 1%	AhR agonist	8	121	7.0 (tapinarof 0.5%), 6.4 (tapinarof 1%), 7.1 (vehicle)	Mild to severe	50.0 (tapinarof 0.5%), 46.3 (tapinarof 1%), 50.0 (vehicle)	11.2 (tapinarof 0.5%), 11.8 (tapinarof 1%), 11.7 (vehicle)	20.2 (tapinarof 0.5%), 20.2 (tapinarof 1%), 20.4 (vehicle)
Eric, 2024 (26)	NCT04773587	US, Canada, Poland	Phase 3	Double-blind, randomized, vehicle-controlled	Mixed adult and pediatric	Roflumilast, QD, 0.15%	PDE4 inhibitor	4	296	NA	Mild to moderate	NA	NA	NA
Eric, 2024 (26)	NCT04773600	US, Canada, Poland	Phase 3	Double-blind, randomized, vehicle-controlled	Mixed adult and pediatric	Roflumilast, QD, 0.15%	PDE4 inhibitor	4	319	NA	Mild to moderate	NA	NA	NA
Hidehisa, 2020 (25)	NCT03018691	Japan	Phase 2	Double-blind, randomized, vehicle-controlled	Pediatric only	Difamilast, BID, 0.3% and 1%	PDE4 inhibitor	4	73	8.5 (difamilast 0.3%), 7.9 (difamilast 1%), 8.5 (vehicle)	Mild to moderate	83.3 (difamilast 0.3%), 80.0 (difamilast 1%), 87.5 (vehicle)	NA	NA
Hidemi, 2019 (23)	JapicCTI-173553	Japan	Phase 2	Double-blind, randomized, vehicle-controlled	Pediatric only	Delgocitinib, BID, 0.25% and 0.5%	JAK inhibitor	4	103	8.5	Mild to severe	57.3	10.94	19.0
Hidemi, 2021 (22)	JapicCTI-184064	Japan	Phase 3	Double-blind, randomized, vehicle-controlled	Pediatric only	Delgocitinib, BID, 0.25%	JAK inhibitor	4	137	8.3	Mild to severe	54.7	10.6	21.2
Lawrence, 2024 (29)	NCT03745638	North America, Europe	Phase 3	Double-blind, randomized, vehicle-controlled	Mixed adult and pediatric	Ruxolitinib, BID, 1.5%	JAK inhibitor	8	70	15.0 (ruxolitinib), 14.0 (vehicle)*	Mild to moderate	75.0 (ruxolitinib), 71.1 (vehicle)	8.0 (ruxolitinib), 8.2 (vehicle)	10.4 (ruxolitinib), 10.8 (vehicle)
Lawrence, 2024 (29)	NCT03745651	North America, Europe	Phase 3	Double-blind, randomized, vehicle-controlled	Mixed adult and pediatric	Ruxolitinib, BID, 1.5%	JAK inhibitor	8	67					
Lawrence, 2025a (19)	NCT04921969	US, Canada	Phase 3	Double-blind, randomized, vehicle-controlled	Pediatric only	Ruxolitinib, BID, 0.75% and 1.5%	JAK inhibitor	8	330	6.0*	Mild to moderate	76.4	8.6	10.5
Lawrence, 2025b (18)	NCT04845620	US, Canada, Poland	Phase 3	Double-blind, randomized, vehicle-controlled	Pediatric only	Roflumilast, QD, 0.05%	PDE4 inhibitor	4	652	3.3 (roflumilast), 3.2 (vehicle)	Mild to moderate	77.3 (roflumilast), 80.0 (vehicle)	11.62 (roflumilast), 12.20 (vehicle)	21.22 (roflumilast), 22.50 (vehicle)
Saeki, 2021 (24)	NCT03911401	Japan	Phase 3	Double-blind, randomized, vehicle-controlled	Pediatric only	Difamilast, BID, 0.3% and 1%	PDE4 inhibitor	4	251	7.1 (difamilast 0.3%), 7.2 (difamilast 1%), 7.1 (vehicle)	Mild to moderate	84.0 (difamilast 0.3%), 84.0 (difamilast 1%), 86.0 (vehicle)	10.8 (difamilast 0.3%), 11.6 (difamilast 1%), 11.3 (vehicle)	NA
Thomas, 2022 (21)	NCT02118766	US	Phase 3	Double-blind, randomized, vehicle-controlled	Mixed adult and pediatric	Crisaborole, BID, 2%	PDE4 inhibitor	4	656	NA	Mild to moderate	61.6 (crisaborole), 64.2 (vehicle)	NA	NA
Thomas, 2022 (21)	NCT02118792	US	Phase 3	Double-blind, randomized, vehicle-controlled	Mixed adult and pediatric	Crisaborole, BID, 2%	PDE4 inhibitor	4	657	NA	Mild to moderate	62.2 (crisaborole), 59.7 (vehicle)	NA	NA
Xiao, 2025 (27)	ChiCTR2200060584	China	Phase 4	Open-label, randomized, active-controlled	Pediatric only	Crisaborole, BID, 2%; pimecrolimus, BID, 1%	PDE4 inhibitor, TCI	4	120	5.32 (crisaborole), 5.63 (pimecrolimus)	Mild to moderate	NA	5.80 (crisaborole), 5.61 (pimecrolimus)	NA
Yan, 2025 (28)	CTR20231413	China	Phase 3	Double-blind, randomized, vehicle-controlled	Mixed adult and pediatric	Tapinarof, BID, 1%	AhR agonist	8	142	NA	Moderate to severe	NA	NA	NA

For trials enrolling both adults and pediatric participants, data were extracted and presented only for the pediatric subgroup when separately available. *Data are presented as medians. AhR, aryl hydrocarbon receptor; BID, twice daily; BSA, body surface area; EASI, Eczema Area and Severity Index; IGA, Investigator’s Global Assessment; JAK, Janus kinase; PDE4, phosphodiesterase-4; QD, once daily.

### 3.2 Primary NMA

**Figure 2:**
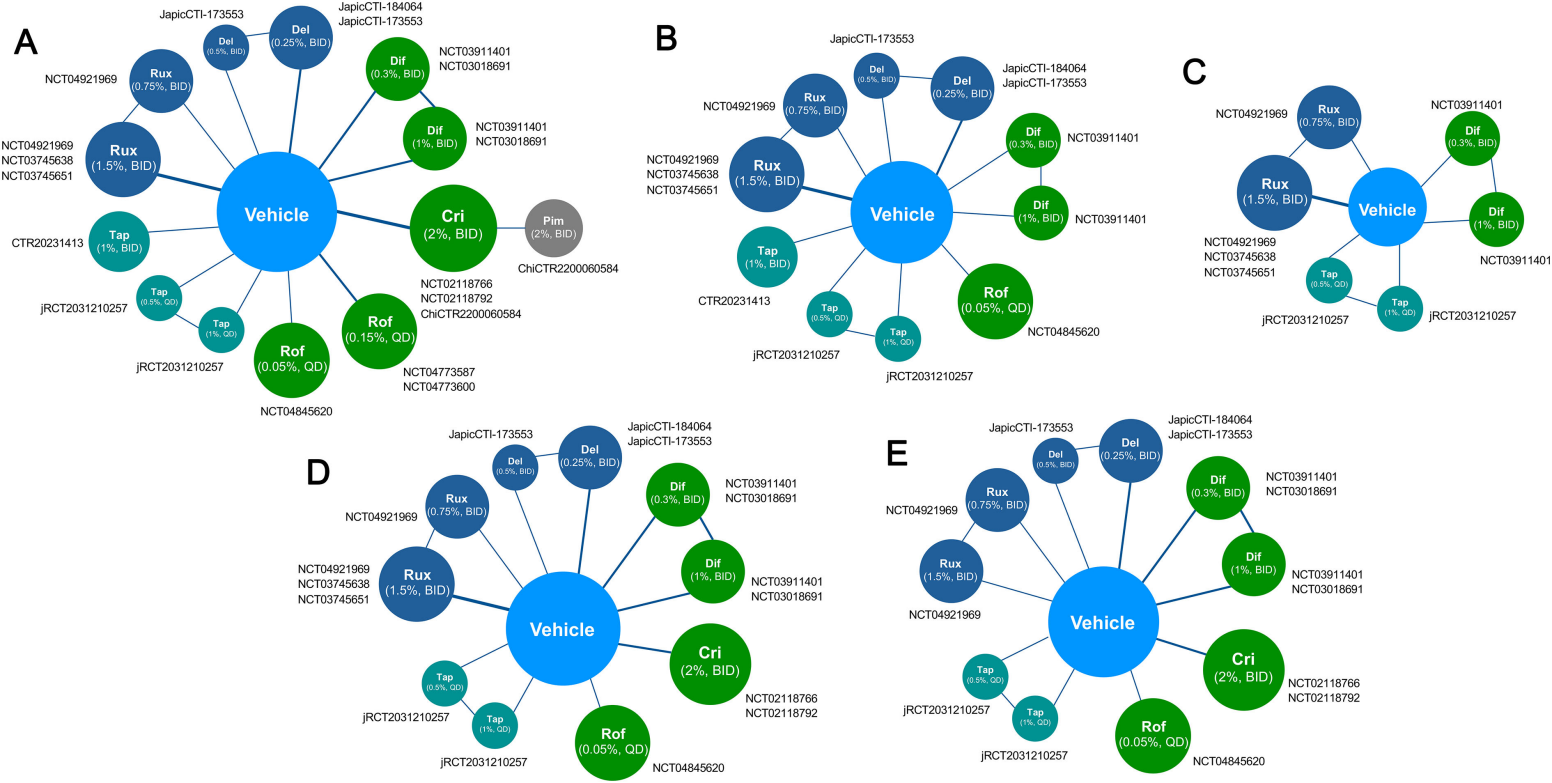
Network plots for the primary network meta-analysis. Network plots are shown for **(A)** IGA-TS, **(B)** EASI-75, **(C)** EASI-90, **(D)** TEAEs, and **(E)** TRAEs. Each node represents an eligible treatment strategy, and node size is proportional to the number of randomized pediatric participants contributing to the corresponding network. The thickness of each line is proportional to the number of randomized controlled trials directly comparing the connected treatment strategies. Clinical trial identifiers are displayed adjacent to each treatment strategy. Treatment labels consist of the abbreviated drug name, concentration, and dosing frequency. Green indicates PDE4 inhibitors, dark blue indicates JAK inhibitors, and cyan-blue indicates AhR agonists. BID, twice daily; Cri, crisaborole; Del, delgocitinib; Dif, difamilast; EASI-75, at least 75% improvement in Eczema Area and Severity Index; EASI-90, at least 90% improvement in Eczema Area and Severity Index; IGA-TS, Investigator’s Global Assessment treatment success; Pim, pimecrolimus; QD, once daily; Rof, roflumilast; Rux, ruxolitinib; Tap, tapinarof; TEAE, treatment-emergent adverse event; TRAE, treatment-related adverse event.

The networks were largely centered on vehicle, which is consistent with the clinical development pathway of these topical agents. For IGA-TS, the network included all 15 trials, 14 treatment nodes, and 3,972 participants. The corresponding networks included 9 trials for EASI-75 response and TRAEs, 5 trials for EASI-90 response, and 11 trials for TEAEs (**Figures 2A–E**). Only one phase 4 trial used an active comparator, evaluating crisaborole against pimecrolimus, direct evidence comparing two active novel nonsteroidal topical agents was currently not available. The transitivity assessment showed that key clinical and methodological characteristics were broadly comparable across the treatment comparisons (**Supplementary Table 3**). In the design-by-treatment interaction model, no statistically significant global inconsistency was detected for IGA-TS, EASI-90, or TRAEs, whereas significant global inconsistency was detected for EASI-75 and TEAEs (**Supplementary Table 4**). Local inconsistency assessed using the separate indirect from direct evidence (SIDE) method under the random-effects model did not detect statistically significant disagreement between direct and indirect evidence in any evaluable comparison (**Supplementary Table 4**).

**Figure 3:**
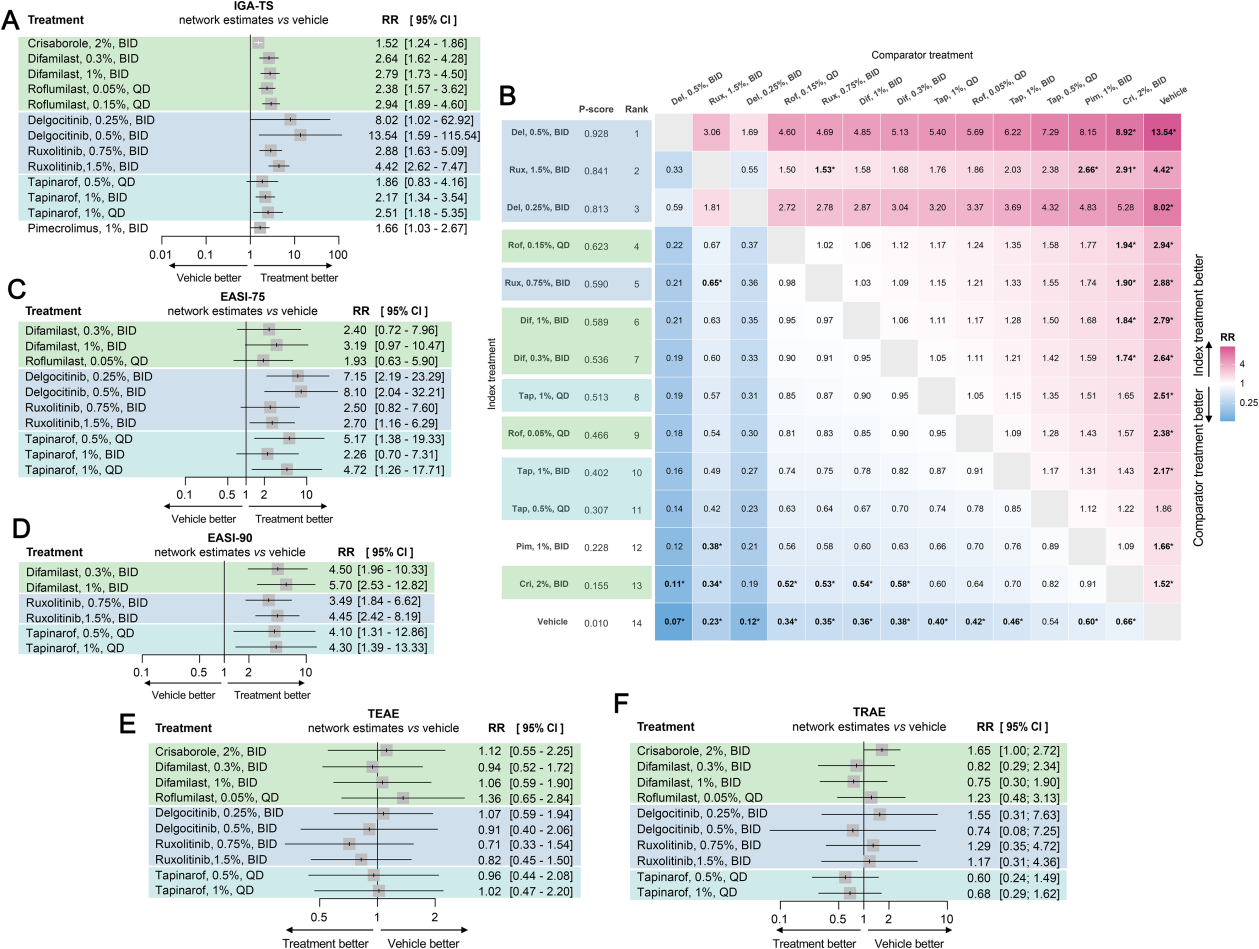
Primary network meta-analysis results for efficacy and safety outcomes. Forest plots are shown for **(A)** IGA-TS, **(C)** EASI-75, **(D)** EASI-90, **(E)** TEAEs, and **(F)** TRAEs, with vehicle as the reference treatment. Risk ratios and 95% confidence intervals are presented for each treatment strategy versus vehicle. For efficacy outcomes, risk ratios greater than 1 favor the active treatment strategy; for safety outcomes, risk ratios greater than 1 indicate a higher incidence of adverse events. **(B)** League-table heatmap for IGA-TS, with treatment strategies ranked by P-score. Each cell shows the risk ratio for the column treatment versus the raw treatment. Heatmap colors are scaled according to log-transformed risk ratios, with values greater than 1 favoring the row treatment for IGA-TS. Legend labels are shown on the original risk ratio scale. *statistically significant. BID, twice daily; Cri, crisaborole; Del, delgocitinib; Dif, difamilast; EASI-75, at least 75% improvement in Eczema Area and Severity Index; EASI-90, at least 90% improvement in Eczema Area and Severity Index; IGA-TS, Investigator’s Global Assessment treatment success; Pim, pimecrolimus; QD, once daily; Rof, roflumilast; Rux, ruxolitinib; Tap, tapinarof; TEAE, treatment-emergent adverse event; TRAE, treatment-related adverse event.

For IGA-TS, most active treatment nodes showed higher response rates than vehicle, although the magnitude and precision of the estimates varied across agents and regimens. Compared with vehicle, tapinarof 0.5% once daily (QD) was the only active node that did not show a statistically significant improvement in IGA-TS (RR, 1.86; 95% CI, 0.83 to 4.16). All other active nodes, including pimecrolimus 1% twice daily (BID), were associated with a significantly higher probability of achieving IGA-TS than vehicle (**Figure 3A**). Furthermore, the league-table heatmap in **Figure 3B** summarizes active-to-active network estimates for IGA-TS together with P-score rankings, providing a comparative overview of the relative efficacy of the evaluated regimens. In active-to-active comparisons, ruxolitinib 1.5% BID showed a significant advantage over pimecrolimus 1% BID (RR = 2.66) and ruxolitinib 0.75% BID (RR = 1.53) (**Figure 3B**; **Supplementary Table 5**). Compared with crisaborole 2% BID, several regimens, including delgocitinib 0.5% BID, difamilast 0.3% and 1% BID, roflumilast 0.15% QD, and ruxolitinib 0.75% and 1.5% BID, were associated with significantly greater IGA-TS benefit (**Figure 3B**; **Supplementary Table 5**). P-score ranking results were broadly consistent with these relative-effect estimates. Delgocitinib 0.5% BID ranked highest for IGA-TS (P-score = 0.928), followed by ruxolitinib 1.5% BID (P-score = 0.841) and delgocitinib 0.25% BID (P-score = 0.813) (**Figure 3B**). Sensitivity analyses excluding the open-label phase 4 active-controlled trial produced results that were generally consistent with the primary analysis (**Supplementary Figure 2**; **Supplementary Table 6**).

For EASI-75 response, all active treatment nodes numerically favored treatment over vehicle, but statistical significance was observed for only five of the ten evaluated active regimens: delgocitinib 0.25% and 0.5% BID, ruxolitinib 1.5% BID, tapinarof 0.5% and 1% QD (**Figure 3C**). The P-score ranking placed delgocitinib 0.5% BID as the most favorable regimen for achieving EASI-75 response (P-score = 0.839), followed by delgocitinib 0.25% BID (P-score = 0.812) and tapinarof 0.5% QD (P-score = 0.704) (**Supplementary Table 7**). For EASI-90 response, the network included 6 treatment regimens across the 3 mechanistic classes, represented by difamilast, ruxolitinib, and tapinarof. All evaluated active regimens showed a significant advantage over vehicle, suggesting that these agents may support deeper clinical improvement in pediatric AD (**Figure 3D**). In the P-score ranking, difamilast 1% BID ranked highest for EASI-90 response (P-score = 0.786), followed by ruxolitinib 1.5% BID (P-score = 0.637) (**Supplementary Table 8**). However, league-table comparisons did not identify any statistically significant difference between active regimens for either EASI-75 or EASI-90 response (**Supplementary Tables 7**, **8**).

For safety outcomes, the primary NMA did not show a consistent increase in TEAEs or TRAEs across active treatment nodes compared with vehicle (**Figures 3E, F**). League-table comparisons likewise showed no statistically significant difference between treatment regimens for either safety outcome (**Supplementary Tables 9**, **10**). In the P-score rankings, ruxolitinib 0.75% BID ranked most favorably for lower TEAE risk (P-score = 0.754), whereas tapinarof 0.5% QD ranked most favorably for lower TRAE risk (P-score = 0.763) (**Supplementary Tables 9**, **10**).

The CINeMA assessment showed variable confidence across the primary active-versus-vehicle network estimates (**Supplementary Table 11**). For IGA-TS, confidence ranged from high to low, with downgrading mainly due to indirectness, imprecision, or heterogeneity. For EASI-90, all evaluated comparisons were rated as moderate confidence because of heterogeneity concerns. Confidence was generally lower for EASI-75, TEAEs, and TRAEs, mainly because of imprecision, heterogeneity, and incoherence signals for EASI-75 and TEAEs.

### 3.3 Subgroup analyses of NMA by treatment duration

**Figure 4:**
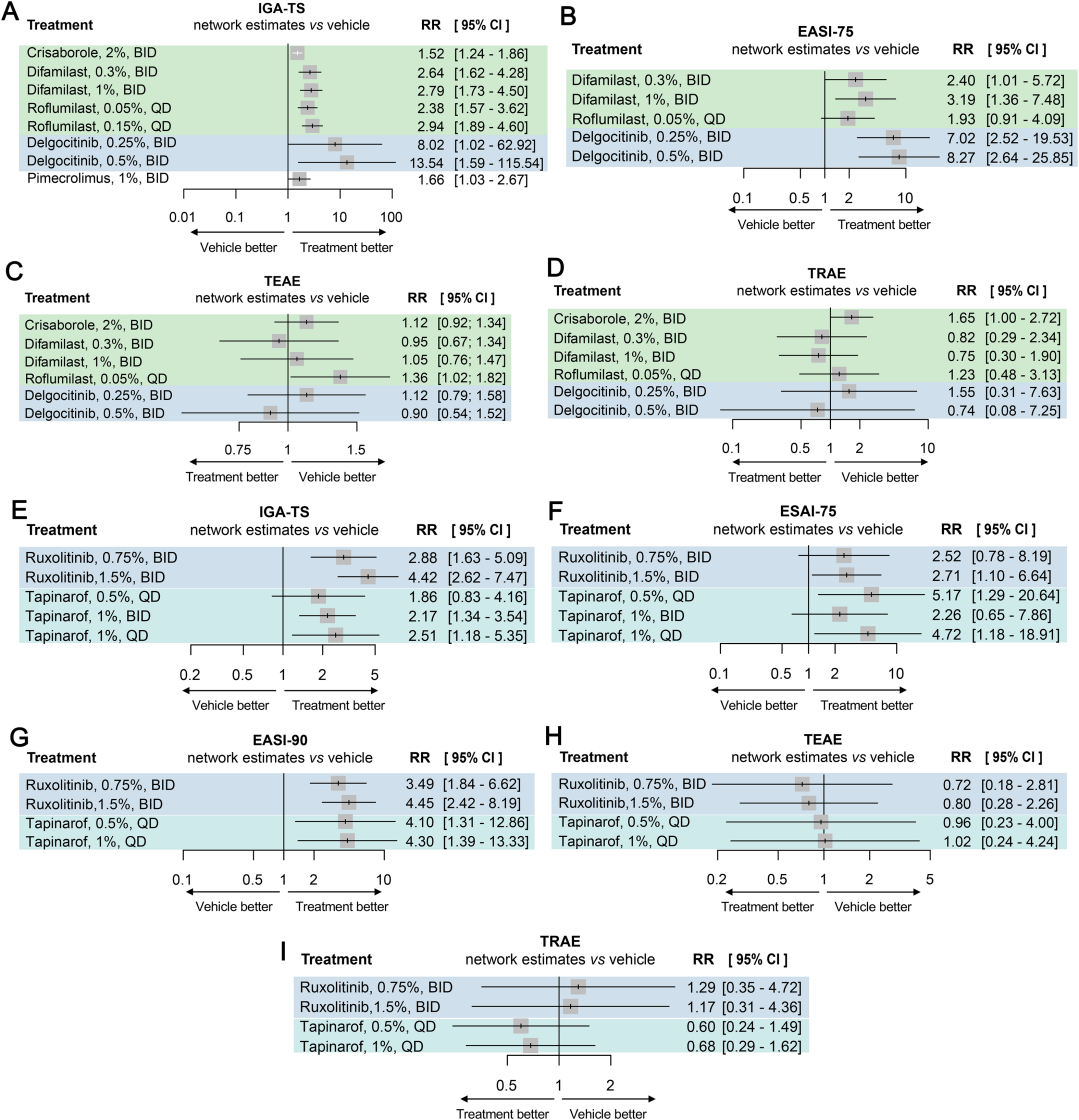
Subgroup network meta-analysis by treatment timepoint. Forest plots are shown for the 4-week subgroup, including **(A)** IGA-TS, **(B)** EASI-75, **(C)** TEAEs, and **(D)** TRAEs, and for the 8-week subgroup, including **(E)** IGA-TS, **(F)** EASI-75, **(G)** EASI-90, **(H)** TEAEs, and **(I)** TRAEs. Risk ratios and 95% confidence intervals are presented for each treatment strategy versus vehicle. For efficacy outcomes, risk ratios greater than 1 favor the active treatment strategy; for safety outcomes, risk ratios greater than 1 indicate a higher incidence of adverse events. BID, twice daily; CI, confidence interval; EASI-75, at least 75% improvement in Eczema Area and Severity Index; EASI-90, at least 90% improvement in Eczema Area and Severity Index; IGA-TS, Investigator’s Global Assessment treatment success; QD, once daily; RR, risk ratio; TEAE, treatment-emergent adverse event; TRAE, treatment-related adverse event.

In the treatment-duration subgroup analysis, trials were stratified according to the end of the randomized treatment period, with separate networks constructed for 4-week and 8-week trials. In the 4-week network, 10 trials and 9 treatment nodes contributed data for IGA-TS, 4 trials contributed data for EASI-75 response, and 7 trials contributed data for TEAEs and TRAEs (**Supplementary Figures 3A–C**). Because only one 4-week trial reported EASI-90 response, NMA was not performed for this endpoint. The 4-week results were generally consistent with the primary analysis (**Figures 4A–D**). All included active treatment nodes showed a significantly higher probability of IGA-TS than vehicle (**Figure 4A**). For EASI-75 response, all evaluated active regimens except roflumilast 0.05% QD showed a statistically significant advantage over vehicle (**Figure 4B**). Short-term safety was broadly comparable between active treatments and vehicle, although roflumilast 0.05% QD was associated with a modestly higher risk of TEAEs (RR, 1.36; 95% CI, 1.02 to 1.82) (**Figures 4C, D**). We performed an additional exploratory analysis that incorporated 4-week outcome data from 8-week trials, when reported, together with trials whose randomized treatment period ended at week 4. In this supplementary analysis, tapinarof 0.5% and 1% QD did not show a clear efficacy advantage over vehicle at week 4 (**Supplementary Figure 4**).

In the 8-week network, 5 trials and 6 treatment nodes contributed data for IGA-TS and EASI-75 response, 4 trials contributed data for EASI-90 response and TEAEs, and 2 trials for TRAEs (**Supplementary Figures 3D–F**). Consistent with the primary analysis, all evaluated active treatment nodes except tapinarof 0.5% QD showed a significant advantage over vehicle for IGA-TS (**Figure 4E**). For EASI-75 response, ruxolitinib 0.75% BID and tapinarof 1% BID did not show statistically significant superiority over vehicle, whereas the remaining active regimens favored treatment (**Figure 4F**). For EASI-90 response, all evaluated active regimens were superior to vehicle (**Figure 4G**). No clear difference in TEAEs or TRAEs was observed between active treatment nodes and vehicle in the 8-week network (**Figures 4H, I**).

### 3.4 Subgroup analyses of NMA by age

**Figure 5:**
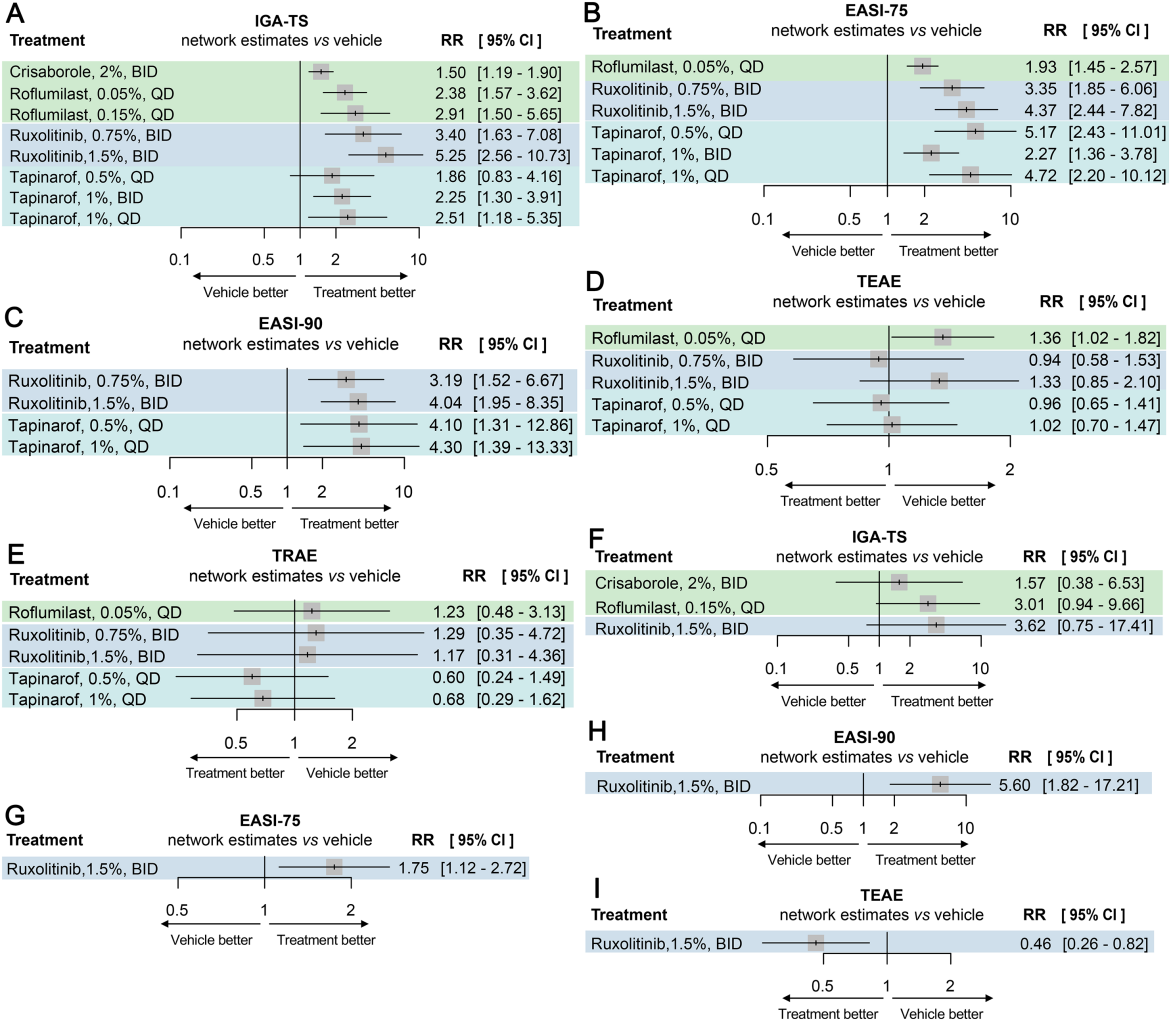
Subgroup network meta-analysis by age group. Forest plots are shown for the subgroup of participants aged <12 years, including **(A)** IGA-TS, **(B)** EASI-75, **(C)** EASI-90, **(D)** TEAEs, and **(E)** TRAEs, and for the subgroup of participants aged ≥12 years, including **(F)** IGA-TS, **(G)** EASI-75, **(H)** EASI-90, and **(I)** TEAEs. Risk ratios and 95% confidence intervals are presented for each treatment strategy versus vehicle. For efficacy outcomes, risk ratios greater than 1 favor the active treatment strategy; for safety outcomes, risk ratios greater than 1 indicate a higher incidence of adverse events. BID, twice daily; CI, confidence interval; EASI-75, at least 75% improvement in Eczema Area and Severity Index; EASI-90, at least 90% improvement in Eczema Area and Severity Index; IGA-TS, Investigator’s Global Assessment treatment success; QD, once daily; RR, risk ratio; TEAE, treatment-emergent adverse event; TRAE, treatment-related adverse event.

Age-based subgroup analyses were conducted separately for children younger than 12 years and those aged 12 years or older. Trials enrolling broad pediatric age ranges were included in the corresponding subgroup networks when age-stratified data were extractable. In the <12-year subgroup, 8 trials and 9 treatment nodes were available for efficacy outcomes, and 3 trials and 6 treatment nodes were available for safety outcomes (**Supplementary Figures 5A–D**). For efficacy, all evaluated active regimens showed a significantly greater benefit than vehicle for IGA-TS, EASI-75 response, or EASI-90 response, except tapinarof 0.5% QD, which did not reach statistical significance for IGA-TS versus vehicle (**Figures 5A-C**). For safety, roflumilast 0.05% QD was associated with a higher risk of TEAEs, whereas no clear increase in adverse events was observed for the other evaluated treatment nodes (**Figures 5D, E**). In the ≥12-year subgroup, 6 trials and 4 treatment nodes contributed data (**Supplementary Figures 5E, F**). The active regimens available for this subgroup were crisaborole 2% BID, roflumilast 0.15% QD, and ruxolitinib 1.5% BID. None of these regimens showed a statistically significant advantage over vehicle for IGA-TS (**Figure 5F**). However, ruxolitinib 1.5% BID was associated with significant benefit for EASI-75 and EASI-90 responses and was also associated with a lower risk of TEAEs compared with vehicle (**Figures 5G–I**).

### 3.5 Pairwise vehicle-controlled meta-analysis

**Figure 6:**
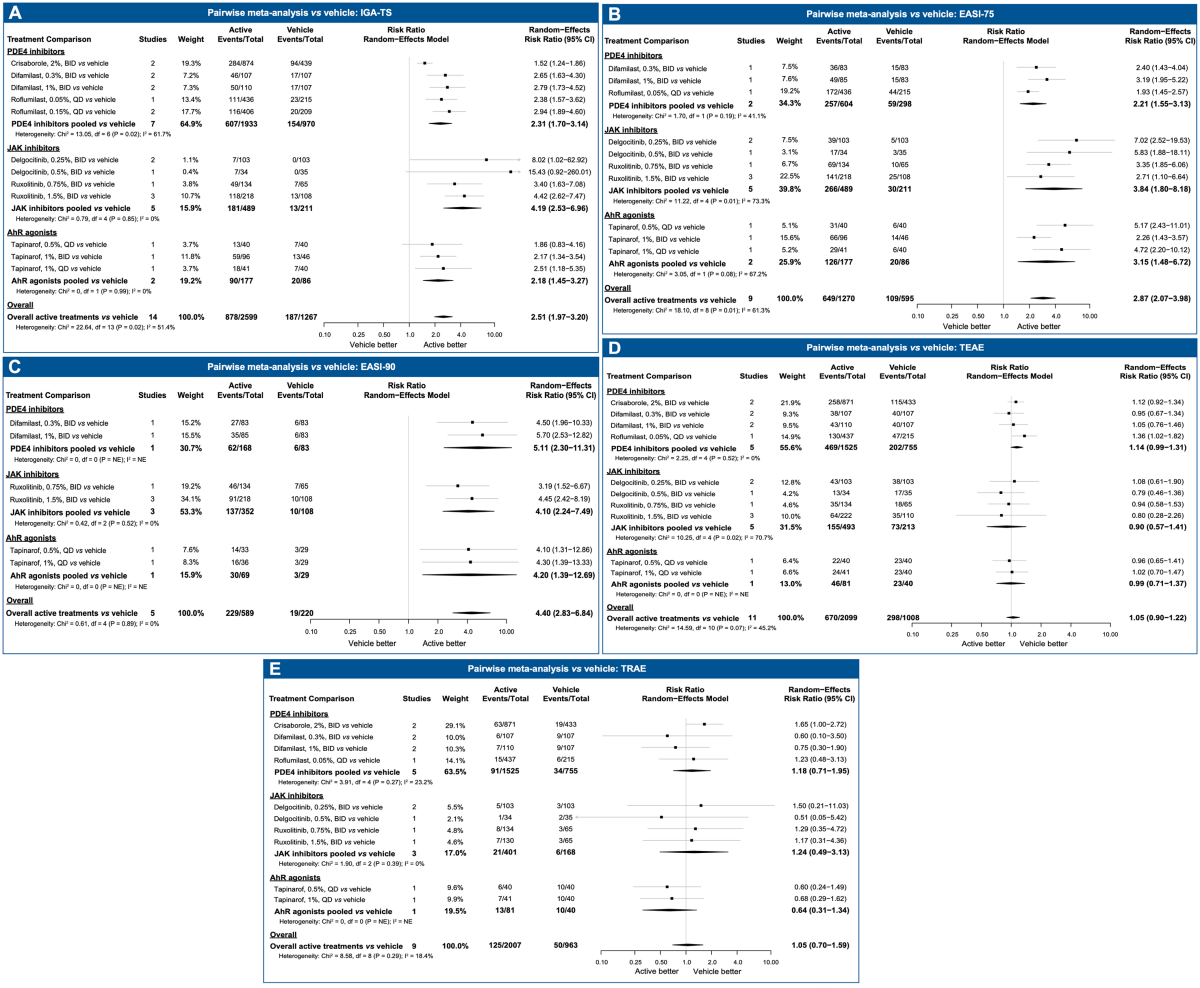
Pairwise meta-analysis of active treatment strategies versus vehicle. Forest plots are shown for **(A)** IGA-TS, **(B)** EASI-75, **(C)** EASI-90, **(D)** TEAEs, and **(E)** TRAEs. Random-effects risk ratios and 95% confidence intervals are presented for each active treatment strategy versus vehicle. Within each outcome, pooled estimates are also shown by mechanistic class and across all active treatment strategies. For efficacy outcomes, risk ratios greater than 1 favor the active treatment strategy; for safety outcomes, risk ratios greater than 1 indicate a higher incidence of adverse events. AhR, aryl hydrocarbon receptor; CI, confidence interval; EASI-75, at least 75% improvement in Eczema Area and Severity Index; EASI-90, at least 90% improvement in Eczema Area and Severity Index; IGA-TS, Investigator’s Global Assessment treatment success; JAK, Janus kinase; PDE4, phosphodiesterase-4; TEAE, treatment-emergent adverse event; TRAE, treatment-related adverse event.

The direct pairwise meta-analysis included all 14 vehicle-controlled trials, comprising 3,866 pediatric patients. Overall, the pairwise findings were consistent with the primary NMA. Compared with vehicle, pooled overall estimates showed that novel nonsteroidal topical therapies had a clear efficacy advantage across the main efficacy endpoints, without a consistent increase in TEAEs or TRAEs (**Figure 6**). At the treatment-regimen level, most active regimens were associated with higher response rates for IGA-TS, EASI-75 response, and EASI-90 response (**Figures 6A–C**). The exceptions were delgocitinib 0.5% BID and tapinarof 0.5% QD for IGA-TS, for which the direct estimates did not reach statistical significance (**Figure 6A**). At the mechanistic class level, PDE4 inhibitors, topical JAK inhibitors, and AhR agonists each favored active treatment over vehicle for efficacy outcomes with similar safety profiles (**Figure 6**).

## 4 Discussion

In this systematic review and NMA of 15 RCTs involving 3,994 pediatric patients with AD, novel nonsteroidal topical therapies showed consistent short-term efficacy advantages over vehicle across the main clinical response outcomes, without a uniform increase in TEAEs or TRAEs. These findings are clinically relevant because topical therapy remains the backbone of pediatric AD management, while novel steroid-sparing agents are increasingly being incorporated into contemporary treatment guidelines alongside moisturizers, TCSs, and TCIs (5, 7). Rather than identifying a single universally preferred treatment, our analysis suggests that the comparative profile of these agents depends on the endpoint, treatment duration, age subgroup, and regimen. These results support the role of novel nonsteroidal topical therapies as evidence-based steroid-sparing options for pediatric AD, while also highlighting the need for more direct comparative, age-stratified, and long-term observational pediatric trials.

Although the overall evidence supported the short-term efficacy of all three mechanistic classes, the response patterns were not identical. The efficacy signal for tapinarof appeared more evident at 8 weeks than at the exploratory 4-week assessment, which is biologically plausible given that AhR activation is linked not only to anti-inflammatory effects, but also to epidermal differentiation, barrier restoration, oxidative stress regulation, and immune homeostasis (13). These processes may require more time to translate into measurable clinical improvement than direct suppression of inflammatory signaling. However, this interpretation should remain cautious because our timepoint analysis was exploratory and was not designed to formally test differences in onset of action. The PDE4 inhibitor class also showed within-class variability, suggesting that agents sharing the same broad target should not be assumed to have interchangeable clinical performance. Differences in PDE4 selectivity, potency, skin penetration, formulation, concentration, dosing schedule, and trial populations may all contribute to the observed variation across crisaborole, difamilast, and roflumilast (30, 31). Topical JAK inhibitors showed favorable efficacy for deeper response outcomes in selected analyses, which is consistent with the central role of JAK-STAT signaling in AD-related cytokine and itch pathways (12, 32). Still, these mechanistic considerations should not be overread as proof of long-term superiority. Our findings are best interpreted as evidence for early clinical response and short-term tolerability.

These findings also have implications for the design of future pediatric AD trials. The current evidence base remains largely organized around vehicle-controlled trials, which are appropriate for establishing initial efficacy but less informative once multiple active topical options are available. As the pediatric topical treatment landscape expands, future development programs should move beyond demonstrating superiority over vehicle alone and generate evidence that better reflects real clinical decision-making. This could include head-to-head trials between novel nonsteroidal topical agents, noninferiority trials against established active comparators, studies designed to test safety advantages or corticosteroid-sparing effects, and pragmatic trials evaluating adherence, treatment satisfaction, and effectiveness in routine care. Longer follow-up is also essential, because short-term clearance or improvement over 4 to 8 weeks does not necessarily predict flare prevention, durability of response, cumulative local tolerability, or safety with repeated intermittent use. Trial populations should also better reflect the children seen in practice, including infants and younger children, patients with involvement of sensitive sites such as the face, eyelids, neck, and flexures, and children across different geographic regions, racial and ethnic backgrounds, and skin tones (33). In addition, pediatric AD drug development should give greater weight to outcomes that matter to children and families, including itch, sleep, school and social functioning, caregiver burden, treatment convenience, out-of-pocket cost, and health-related quality of life (34, 35). These patient- and family-centered endpoints are not secondary concerns; they are central to judging whether a topical therapy is meaningfully useful in a chronic, relapsing childhood disease.

To our knowledge, it provides the first pediatric-focused NMA comparisons of novel nonsteroidal topical therapies for AD across PDE4 inhibitors, topical JAK inhibitors, and AhR agonists. By integrating both direct vehicle-controlled evidence and indirect network comparisons, this analysis offers a structured overview of the relative short-term efficacy and tolerability of currently available and emerging topical regimens in children. The use of regimen-level treatment nodes also allowed clinically relevant differences in concentration and dosing frequency to be preserved, rather than assuming that agents within the same mechanistic class are interchangeable. In addition, subgroup analyses by treatment duration and age provided a more granular view of how the available evidence applies to different pediatric settings. Several limitations should also be acknowledged. First, although the analysis included 15 randomized trials, the evidence network remained relatively sparse for several outcomes, particularly EASI-90 response, TRAEs, and age-stratified analyses. Second, all these trials were conducted in North America, Europe, and East Asia, which may limit the generalizability of the findings to children from other geographic regions, racial and ethnic backgrounds, and skin tones. Third, age distributions, baseline disease severity, treatment duration, and outcome assessment time points differed across studies, and these differences may have influenced the transitivity assumption required for NMA. As a result, treatment rankings should be interpreted cautiously and should not be viewed as definitive proof of superiority. Furthermore, the CINeMA assessment supports a cautious interpretation of the comparative findings. Although several IGA-TS comparisons were supported by high or moderate confidence, many EASI-75, TEAE, and TRAE estimates were rated as low or very low confidence, mainly because of imprecision, heterogeneity, and incoherence signals. These findings further highlight the need for well-designed head-to-head trials and long-term real-world data to clarify comparative effectiveness and safety in pediatric AD. Finally, the included randomized treatment periods were limited to 4 to 8 weeks. For a chronic and relapsing disease such as pediatric AD, these data are informative for early efficacy and short-term tolerability, but they cannot directly determine durability of response, flare prevention, long-term adherence, cumulative local tolerability, or safety with repeated intermittent use.

## 5 Conclusion

Novel nonsteroidal topical therapies, including PDE4 inhibitors, topical JAK inhibitors, and AhR agonists, showed consistent short-term efficacy advantages over vehicle in pediatric AD, without a clear overall increase in TEAEs or TRAEs. The comparative profiles of these agents differed across endpoints, treatment duration, age subgroup, and regimen, suggesting that pediatric topical treatment selection should consider more than drug class alone. However, the current evidence remains largely based on short-term vehicle-controlled trials, with limited direct active-comparator evidence and sparse age-stratified data. These findings support the role of novel nonsteroidal topical agents as steroid-sparing options for pediatric AD while underscoring the need for head-to-head trials, longer follow-up, and more patient- and family-centered evidence to guide real-world treatment decisions.

## Data Availability

The original contributions presented in the study are included in the article/**Supplementary Material**. Further inquiries can be directed to the corresponding authors.
