# Supplementary material for: Novel nonsteroidal topical therapies for pediatric atopic dermatitis: a systematic review and network meta-analysis of randomized controlled trials
Source: Front Immunol. 2026 Jul 22;17:1906218. doi: 10.3389/fimmu.2026.1906218 (PMC13438444; doi:10.3389/fimmu.2026.1906218)
Supplement: Supplementary file 1 [file DataSheet1.docx]

**Novel Nonsteroidal Topical Therapies for Pediatric Atopic Dermatitis: A Systematic Review and Network Meta-analysis of Randomized Controlled Trials**

Supplement Material

**Content**

[Supplement Table 1. PRISMA NMA Checklist.	3](#_Toc233320883)

[Supplementary Table 2. Detailed search strategies.	7](#_Toc233320884)

[Supplementary Table 3. Summary of transitivity assessment across treatment regimen.	12](#_Toc233320885)

[Supplementary Table 4. Global and local inconsistency assessments.	14](#_Toc233320886)

[Supplementary Table 5. P-scores, treatment rankings, and league table for IGA treatment success in the primary network meta-analysis.	15](#_Toc233320887)

[Supplement Table 6. P-scores, treatment rankings, and league table for IGA treatment success in the sensitivity analysis.	17](#_Toc233320888)

[Supplementary Table 7. P-scores, treatment rankings, and league table for EASI-75 in the primary network meta-analysis.	20](#_Toc233320889)

[Supplementary Table 8. P-scores, treatment rankings, and league table for EASI-90 in the primary network meta-analysis.	22](#_Toc233320890)

[Supplementary Table 9. P-scores, treatment rankings, and league table for TEAE in the primary network meta-analysis.	23](#_Toc233320891)

[Supplementary Table 10. P-scores, treatment rankings, and league table for TRAE in the primary network meta-analysis.	24](#_Toc233320892)

[Supplementary Table 11. CINeMA assessment of confidence in the primary active-versus-vehicle network estimates.	26](#_Toc233320893)

[Supplementary Figure 1. Traffic light plot of study-level risk-of-bias assessment using the Cochrane RoB 2 tool.	32](#_Toc233320894)

[Supplementary Figure 2. Sensitivity analysis of the primary network meta-analysis for IGA treatment success.	33](#_Toc233320895)

[Supplementary Figure 3. Network plots of the timepoint subgroup analyses.	34](#_Toc233320896)

[Supplementary Figure 4. Supplemental week-4 efficacy analysis including trials with reported week-4 outcomes.	35](#_Toc233320897)

[Supplementary Figure 5. Network plots for the age subgroup analyses.	36](#_Toc233320898)

# Supplement Table 1. PRISMA NMA Checklist.

| **Section/Topic** | **Item #** | **Checklist Item** | **Reported on Page #** |
| --- | --- | --- | --- |
| **TITLE** |  |  |  |
| Title | 1 | Identify the report as a systematic review *incorporating a network meta-analysis (or related form of meta-analysis).* | ***Page 1*** |
|  |  |  |  |
| **ABSTRACT** |  |  |  |
| Structured summary | 2 | Provide a structured summary including, as applicable:  **Background:** main objectives  **Methods:** data sources; study eligibility criteria, participants, and interventions; study appraisal; and *synthesis methods, such as network meta-analysis.*  **Results:** number of studies and participants identified; summary estimates with corresponding confidence/credible intervals; *treatment rankings may also be discussed. Authors may choose to summarize pairwise comparisons against a chosen treatment included in their analyses for brevity.*  **Discussion/Conclusions:** limitations; conclusions and implications of findings.  **Other:** primary source of funding; systematic review registration number with registry name. | Page 2 |
|  |  |  |  |
| **INTRODUCTION** |  |  |  |
| Rationale | 3 | Describe the rationale for the review in the context of what is already known*, including mention of why a network meta-analysis has been conducted.* | ***Page 3-4*** |
| Objectives | 4 | Provide an explicit statement of questions being addressed, with reference to participants, interventions, comparisons, outcomes, and study design (PICOS). | Page 4-5 |
|  |  |  |  |
| **METHODS** |  |  |  |
| Protocol and registration | 5 | Indicate whether a review protocol exists and if and where it can be accessed (e.g., Web address); and, if available, provide registration information, including registration number. | Page 5 |
| Eligibility criteria | 6 | Specify study characteristics (e.g., PICOS, length of follow-up) and report characteristics (e.g., years considered, language, publication status) used as criteria for eligibility, giving rationale. *Clearly describe eligible treatments included in the treatment network, and note whether any have been clustered or merged into the same node (with justification).* | ***Page 5-6*** |
| Information sources | 7 | Describe all information sources (e.g., databases with dates of coverage, contact with study authors to identify additional studies) in the search and date last searched. | ***Page 5-6*** |
| Search | 8 | Present full electronic search strategy for at least one database, including any limits used, such that it could be repeated. | Supplementary Table 2 |
| Study selection | 9 | State the process for selecting studies (i.e., screening, eligibility, included in systematic review, and, if applicable, included in the meta-analysis). | Figure 1 |
| Data collection process | 10 | Describe method of data extraction from reports (e.g., piloted forms, independently, in duplicate) and any processes for obtaining and confirming data from investigators. | Page 6-7 |
| Data items | 11 | List and define all variables for which data were sought (e.g., PICOS, funding sources) and any assumptions and simplifications made. | Page 6 |
| **Geometry of the network** | **S1** | Describe methods used to explore the geometry of the treatment network under study and potential biases related to it. This should include how the evidence base has been graphically summarized for presentation, and what characteristics were compiled and used to describe the evidence base to readers. | ***Page 7*** |
| Risk of bias within individual studies | 12 | Describe methods used for assessing risk of bias of individual studies (including specification of whether this was done at the study or outcome level), and how this information is to be used in any data synthesis. | Page 7 |
| Summary measures | 13 | State the principal summary measures (e.g., risk ratio, difference in means). *Also describe the use of additional summary measures assessed, such as treatment rankings and surface under the cumulative ranking curve (SUCRA) values, as well as modified approaches used to present summary findings from meta-analyses.* | Page 7-8 |
| Planned methods of analysis | 14 | Describe the methods of handling data and combining results of studies for each network meta-analysis. This should include, but not be limited to:   - *Handling of multi-arm trials;* - *Selection of variance structure;* - *Selection of prior distributions in Bayesian analyses; and* - *Assessment of model fit.* | Page 7-8 |
| **Assessment of Inconsistency** | **S2** | Describe the statistical methods used to evaluate the agreement of direct and indirect evidence in the treatment network(s) studied. Describe efforts taken to address its presence when found. | Page 7 |
| Risk of bias across studies | 15 | Specify any assessment of risk of bias that may affect the cumulative evidence (e.g., publication bias, selective reporting within studies). | **Page 7** |
| Additional analyses | 16 | Describe methods of additional analyses if done, indicating which were pre-specified. This may include, but not be limited to, the following:   - Sensitivity or subgroup analyses; - Meta-regression analyses; - *Alternative formulations of the treatment network; and* - *Use of alternative prior distributions for Bayesian analyses (if applicable).* | ***Page 7-8*** |
|  |  |  |  |
| **RESULTS†** |  |  |  |
| Study selection | 17 | Give numbers of studies screened, assessed for eligibility, and included in the review, with reasons for exclusions at each stage, ideally with a flow diagram. | Page 9 and Figure 1 |
| **Presentation of network structure** | **S3** | Provide a network graph of the included studies to enable visualization of the geometry of the treatment network. | ***Figure 2, Supplementary Figure 2-4*** |
| **Summary of network geometry** | **S4** | Provide a brief overview of characteristics of the treatment network. This may include commentary on the abundance of trials and randomized patients for the different interventions and pairwise comparisons in the network, gaps of evidence in the treatment network, and potential biases reflected by the network structure. | ***Page 9-13*** |
| Study characteristics | 18 | For each study, present characteristics for which data were extracted (e.g., study size, PICOS, follow-up period) and provide the citations. | Table 1 |
| Risk of bias within studies | 19 | Present data on risk of bias of each study and, if available, any outcome level assessment. | Supplementary Figure 1 |
| Results of individual studies | 20 | For all outcomes considered (benefits or harms), present, for each study: 1) simple summary data for each intervention group, and 2) effect estimates and confidence intervals. *Modified approaches may be needed to deal with information from larger networks.* | ***Figure 3*** |
| Synthesis of results | 21 | Present results of each meta-analysis done, including confidence/credible intervals. *In larger networks, authors may focus on comparisons versus a particular comparator (e.g. placebo or standard care), with full findings presented in an appendix. League tables and forest plots may be considered to summarize pairwise comparisons.* If additional summary measures were explored (such as treatment rankings), these should also be presented. | ***Figure 3, Supplementary Table 3-8*** |
| **Exploration for inconsistency** | **S5** | Describe results from investigations of inconsistency. This may include such information as measures of model fit to compare consistency and inconsistency models, *P* values from statistical tests, or summary of inconsistency estimates from different parts of the treatment network. | ***Page 9*** |
| Risk of bias across studies | 22 | Present results of any assessment of risk of bias across studies for the evidence base being studied. | Page 9, Supplementary Figure 1 |
| Results of additional analyses | 23 | Give results of additional analyses, if done (e.g., sensitivity or subgroup analyses, meta-regression analyses*, alternative network geometries studied, alternative choice of prior distributions for Bayesian analyses,* and so forth). | ***Page 10-13, Supplementary Figure 2 and 4, Figure 4-6*** |
|  |  |  |  |
| **DISCUSSION** |  |  |  |
| Summary of evidence | 24 | Summarize the main findings, including the strength of evidence for each main outcome; consider their relevance to key groups (e.g., healthcare providers, users, and policy-makers). | Page 13-14 |
| Limitations | 25 | Discuss limitations at study and outcome level (e.g., risk of bias), and at review level (e.g., incomplete retrieval of identified research, reporting bias). *Comment on the validity of the assumptions, such as transitivity and consistency. Comment on any concerns regarding network geometry (e.g., avoidance of certain comparisons).* | Page 16 |
| Conclusions | 26 | Provide a general interpretation of the results in the context of other evidence, and implications for future research. | Page 16-17 |
|  |  |  |  |
| **FUNDING** |  |  |  |
| Funding | 27 | Describe sources of funding for the systematic review and other support (e.g., supply of data); role of funders for the systematic review. This should also include information regarding whether funding has been received from manufacturers of treatments in the network and/or whether some of the authors are content experts with professional conflicts of interest that could affect use of treatments in the network. | ***Page 16*** |

PICOS = population, intervention, comparators, outcomes, study design.

# Supplementary Table 2. Detailed search strategies.

| Database | Search strategy | Records |
| --- | --- | --- |
| PubMed | ("Dermatitis, Atopic"[Mesh] OR "atopic dermatitis"[tiab] OR eczema[tiab] OR "atopic eczema"[tiab])  AND  (child*[tiab] OR pediatric*[tiab] OR paediatric*[tiab] OR adolescent*[tiab] OR infant*[tiab] OR toddler*[tiab] OR "Child"[Mesh] OR "Infant"[Mesh] OR "Adolescent"[Mesh])  AND  (topical[tiab] OR cream[tiab] OR ointment[tiab] OR "topical therapy"[tiab] OR "Administration, Topical"[Mesh])  AND  (nonsteroidal[tiab] OR non-steroidal[tiab] OR "steroid-sparing"[tiab] OR "PDE4 inhibitor"[tiab] OR "phosphodiesterase 4 inhibitor"[tiab] OR "JAK inhibitor"[tiab] OR "Janus kinase inhibitor"[tiab] OR "AhR agonist"[tiab] OR "aryl hydrocarbon receptor"[tiab] OR crisaborole[tiab] OR roflumilast[tiab] OR difamilast[tiab] OR "OPA-15406"[tiab] OR ruxolitinib[tiab] OR delgocitinib[tiab] OR "JTE-052"[tiab] OR tapinarof[tiab] OR "GSK2894512"[tiab])  AND  (randomized[tiab] OR randomised[tiab] OR placebo[tiab] OR vehicle[tiab] OR "double-blind"[tiab] OR "controlled trial"[tiab] OR "Randomized Controlled Trial"[Publication Type]) | 132 |
| Embase | ('atopic dermatitis'/exp OR 'atopic dermatitis':ti,ab,kw OR eczema:ti,ab,kw OR 'atopic eczema':ti,ab,kw) AND ('child'/exp OR 'infant'/exp OR 'adolescent'/exp OR child*:ti,ab,kw OR pediatric*:ti,ab,kw OR paediatric*:ti,ab,kw OR adolescent*:ti,ab,kw OR infant*:ti,ab,kw OR toddler*:ti,ab,kw) AND ('topical drug administration'/exp OR topical:ti,ab,kw OR cream:ti,ab,kw OR creams:ti,ab,kw OR ointment:ti,ab,kw OR ointments:ti,ab,kw OR 'topical therapy':ti,ab,kw OR 'topical treatment':ti,ab,kw OR 'topical application':ti,ab,kw) AND (nonsteroidal:ti,ab,kw OR 'non-steroidal':ti,ab,kw OR 'steroid sparing':ti,ab,kw OR 'steroid-sparing':ti,ab,kw OR 'phosphodiesterase 4 inhibitor'/exp OR 'pde4 inhibitor':ti,ab,kw OR 'pde-4 inhibitor':ti,ab,kw OR 'phosphodiesterase 4 inhibitor':ti,ab,kw OR 'phosphodiesterase-4 inhibitor':ti,ab,kw OR 'janus kinase inhibitor'/exp OR 'jak inhibitor':ti,ab,kw OR 'janus kinase inhibitor':ti,ab,kw OR 'aryl hydrocarbon receptor agonist':ti,ab,kw OR 'ahr agonist':ti,ab,kw OR 'aryl hydrocarbon receptor':ti,ab,kw OR 'crisaborole'/exp OR crisaborole:ti,ab,kw OR 'roflumilast'/exp OR roflumilast:ti,ab,kw OR 'difamilast'/exp OR difamilast:ti,ab,kw OR 'opa-15406':ti,ab,kw OR 'ruxolitinib'/exp OR ruxolitinib:ti,ab,kw OR 'delgocitinib'/exp OR delgocitinib:ti,ab,kw OR 'jte-052':ti,ab,kw OR 'tapinarof'/exp OR tapinarof:ti,ab,kw OR 'gsk2894512':ti,ab,kw) AND ('randomized controlled trial'/de OR 'controlled clinical trial'/de OR 'randomization'/exp OR 'double blind procedure'/exp OR 'single blind procedure'/exp OR 'placebo'/exp OR randomized:ti,ab,kw OR randomised:ti,ab,kw OR randomly:ti,ab,kw OR placebo:ti,ab,kw OR vehicle:ti,ab,kw OR 'double blind':ti,ab,kw OR 'double-blind':ti,ab,kw OR 'single blind':ti,ab,kw OR 'single-blind':ti,ab,kw OR 'controlled trial':ti,ab,kw OR 'clinical trial':ti,ab,kw) | 480 |
| Cochrane Library | #1 MeSH descriptor: [Dermatitis, Atopic] explode all trees  #2 ("atopic dermatitis" OR eczema OR "atopic eczema"):ti,ab,kw  #3 #1 OR #2  #4 MeSH descriptor: [Child] explode all trees  #5 MeSH descriptor: [Infant] explode all trees  #6 MeSH descriptor: [Adolescent] explode all trees  #7 (child* OR pediatric* OR paediatric* OR adolescent* OR infant* OR toddler*):ti,ab,kw  #8 #4 OR #5 OR #6 OR #7  #9 MeSH descriptor: [Administration, Topical] explode all trees  #10 (topical OR cream* OR ointment* OR "topical therapy" OR "topical treatment" OR "topical application"):ti,ab,kw  #11 #9 OR #10  #12 MeSH descriptor: [Phosphodiesterase 4 Inhibitors] explode all trees  #13 MeSH descriptor: [Janus Kinase Inhibitors] explode all trees  #14 MeSH descriptor: [Receptors, Aryl Hydrocarbon] explode all trees  #15 (  nonsteroidal OR "non-steroidal" OR "steroid sparing" OR "steroid-sparing"  OR "PDE4 inhibitor" OR "PDE-4 inhibitor" OR "phosphodiesterase 4 inhibitor" OR "phosphodiesterase-4 inhibitor"  OR "JAK inhibitor" OR "Janus kinase inhibitor"  OR "AhR agonist" OR "aryl hydrocarbon receptor"  OR crisaborole OR roflumilast OR difamilast OR "OPA-15406"  OR ruxolitinib OR delgocitinib OR "JTE-052"  OR tapinarof OR "GSK2894512"  ):ti,ab,kw  #16 #12 OR #13 OR #14 OR #15  #17 #3 AND #8 AND #11 AND #16 | 331 |
| Web of Science | TS=(  "atopic dermatitis"  OR eczema  OR "atopic eczema"  )  AND  TS=(  child*  OR pediatric*  OR paediatric*  OR adolescent*  OR infant*  OR toddler*  )  AND  TS=(  topical  OR cream*  OR ointment*  OR "topical therapy"  OR "topical treatment"  OR "topical application"  )  AND  TS=(  nonsteroidal  OR "non-steroidal"  OR "steroid sparing"  OR "steroid-sparing"  OR "PDE4 inhibitor"  OR "PDE-4 inhibitor"  OR "phosphodiesterase 4 inhibitor"  OR "phosphodiesterase-4 inhibitor"  OR "JAK inhibitor"  OR "Janus kinase inhibitor"  OR "AhR agonist"  OR "aryl hydrocarbon receptor"  OR crisaborole  OR roflumilast  OR difamilast  OR "OPA-15406"  OR ruxolitinib  OR delgocitinib  OR "JTE-052"  OR tapinarof  OR "GSK2894512"  )  AND  TS=(  randomized  OR randomised  OR randomly  OR placebo  OR vehicle  OR "double blind"  OR "double-blind"  OR "single blind"  OR "single-blind"  OR "controlled trial"  OR "clinical trial"  ) | 229 |

The search strategies were adapted for each database using database-specific controlled vocabulary and free-text terms related to atopic dermatitis, pediatric populations, topical therapy, and novel nonsteroidal anti-inflammatory agents. Searches were conducted from database inception to May 31, 2026, with no restrictions on publication year or language.

# Supplementary Table 3. Summary of transitivity assessment across treatment regimen.

| Treatment | Trial | Study phase | Control type | Blinding status | Age range, years | Baseline IGA severity | Treatment duration, weeks | Outcome assessment time point, week |
| --- | --- | --- | --- | --- | --- | --- | --- | --- |
| Crisaborole, 2%, BID | NCT02118766, NCT02118792, ChiCTR2200060584 | 3, 4 | Active control, vehicle control | Double blind, open label | 2 to 17 | Mild to moderate | 4 | 4 |
| Delgocitinib, 0.25%, BID | JapicCTI-184064, JapicCTI-173553 | 2, 3 | Vehicle control | Double blind | 2 to 15 | Mild to severe | 4 | 4 |
| Delgocitinib, 0.5%, BID | JapicCTI-173553 | 2 | Vehicle control | Double blind | 2 to 15 | Mild to severe | 4 | 4 |
| Difamilast, 0.3%, BID | NCT03911401, NCT03018691 | 2, 3 | Vehicle control | Double blind | 2 to 14 | Mild to moderate | 4 | 4 |
| Difamilast, 1%, BID | NCT03911401, NCT03018691 | 2, 3 | Vehicle control | Double blind | 2 to 14 | Mild to moderate | 4 | 4 |
| Pimecrolimus, 1%, BID | ChiCTR2200060584 | 4 | Active control | Open label | 2 to 17 | Mild to moderate | 4 | 4 |
| Roflumilast, 0.05%, QD | NCT04845620 | 3 | Vehicle control | Double blind | 2 to 5 | Mild to moderate | 4 | 4 |
| Roflumilast, 0.15%, QD | NCT04773587, NCT04773600 | 3 | Vehicle control | Double blind | 6 to 17 | Mild to moderate | 4 | 4 |
| Ruxolitinib, 0.75%, BID | NCT04921969 | 3 | Vehicle control | Double blind | 2 to 11 | Mild to moderate | 8 | 4, 8 |
| Ruxolitinib, 1.5%, BID | NCT04921969, NCT03745638, NCT03745651 | 3 | Vehicle control | Double blind | 2 to 17 | Mild to moderate | 8 | 4, 8 |
| Tapinarof, 0.5%, QD | jRCT2031210257 | 2 | Vehicle control | Double blind | 2 to 11 | Mild to severe | 8 | 4, 8 |
| Tapinarof, 1%, BID | CTR20231413 | 3 | Vehicle control | Double blind | 2 to 17 | Moderate to severe | 8 | 8 |
| Tapinarof, 1%, QD | jRCT2031210257 | 2 | Vehicle control | Double blind | 2 to 11 | Mild to severe | 8 | 4, 8 |
| Vehicle | NCT02118766, NCT02118792, JapicCTI-184064, JapicCTI-173553, NCT03911401, NCT03018691, NCT04845620, NCT04773587, NCT04773600, NCT04921969, NCT03745638, NCT03745651, jRCT2031210257, CTR20231413 | 2, 3 | Vehicle control | Double blind | 2 to 17 | Mild to severe | 4, 8 | 4, 8 |

BID, twice daily; IGA, Investigator’s Global Assessment; QD, once daily.

# Supplementary Table 4. Global and local inconsistency assessments.

| Endpoint | Global inconsistency, between-designs Q | df | Global *P-*value | Local inconsistency, SIDE random-effects model | Local *P*-value range | Interpretation |
| --- | --- | --- | --- | --- | --- | --- |
| IGA-TS | 0.49 | 2 | 0.781 | 6 evaluable comparisons | 0.491 to 0.889 | No statistically significant global or local inconsistency detected |
| EASI-75 | 7.33 | 2 | 0.026 | 4 evaluable comparisons | 0.289 to 0.384 | Significant global inconsistency detected, but no statistically significant local inconsistency under the random-effects SIDE model |
| EASI-90 | 0.23 | 1 | 0.632 | 2 evaluable comparisons | 0.632 | No statistically significant global or local inconsistency detected |
| TEAE | 10.6 | 2 | 0.005 | 4 evaluable comparisons with formal P values; 2 comparisons not formally estimable | 0.084 to 0.338 | Significant global inconsistency detected, but no statistically significant local inconsistency under the random-effects SIDE model |
| TRAE | 1.39 | 1 | 0.238 | 4 evaluable comparisons | 0.212 to 0.569 | No statistically significant global or local inconsistency detected |

Global inconsistency was assessed using the design-by-treatment interaction model. The reported Q statistic refers to the between-designs Q statistic. Local inconsistency was assessed using the separate indirect from direct evidence (SIDE) approach under the random-effects model. The local *P*-value range summarizes evaluable comparisons only. A *P*-value <0.05 indicates statistically significant evidence of inconsistency. EASI-75, at least 75% improvement in Eczema Area and Severity Index; EASI-90, at least 90% improvement in Eczema Area and Severity Index; IGA-TS, Investigator’s Global Assessment treatment success; SIDE, separate indirect from direct evidence; TEAE, treatment-emergent adverse event; TRAE, treatment-related adverse event.

# Supplementary Table 5. P-scores, treatment rankings, and league table for IGA treatment success in the primary network meta-analysis.

| P-score | Rank | Treatment | Delgocitinib, 0.5%, BID | Ruxolitinib, 1.5%, BID | Delgocitinib, 0.25%, BID | Roflumilast, 0.15%, QD | Ruxolitinib, 0.75%, BID | Difamilast, 1%, BID | Difamilast, 0.3%, BID | Tapinarof, 1%, QD | Roflumilast, 0.05%, QD | Tapinarof, 1%, BID | Tapinarof, 0.5%, QD | Pimecrolimus, 1%, BID | Crisaborole, 2%, BID | Vehicle |
| --- | --- | --- | --- | --- | --- | --- | --- | --- | --- | --- | --- | --- | --- | --- | --- | --- |
| 0.928 | 1 | Delgocitinib, 0.5%, BID |  | 3.06 (0.34-27.84) | 1.69 (0.59-4.84) | 4.60 (0.51-41.09) | 4.69 (0.51-43.15) | 4.85 (0.54-43.68) | 5.13 (0.57-46.26) | 5.40 (0.56-52.43) | 5.69 (0.64-50.55) | 6.22 (0.69-56.11) | 7.29 (0.74-72.07) | 8.15 (0.91-73.27) | 8.92 (1.04-76.87)* | 13.54 (1.59-115.54)* |
| 0.841 | 2 | Ruxolitinib, 1.5%, BID | 0.33 (0.04-2.97) |  | 0.55 (0.07-4.62) | 1.50 (0.75-2.99) | 1.53 (1.17-2.00)* | 1.58 (0.78-3.23) | 1.68 (0.82-3.43) | 1.76 (0.70-4.42) | 1.86 (0.95-3.63) | 2.03 (0.99-4.16) | 2.38 (0.91-6.23) | 2.66 (1.31-5.40)* | 2.91 (1.66-5.11)* | 4.42 (2.62-7.47)* |
| 0.813 | 3 | Delgocitinib, 0.25%, BID | 0.59 (0.21-1.70) | 1.81 (0.22-15.20) |  | 2.72 (0.33-22.42) | 2.78 (0.33-23.57) | 2.87 (0.35-23.84) | 3.04 (0.37-25.25) | 3.20 (0.36-28.69) | 3.37 (0.41-27.58) | 3.69 (0.44-30.62) | 4.32 (0.47-39.47) | 4.83 (0.58-39.99) | 5.28 (0.67-41.88) | 8.02 (1.02-62.92)* |
| 0.623 | 4 | Roflumilast, 0.15%, QD | 0.22 (0.02-1.94) | 0.67 (0.33-1.33) | 0.37 (0.04-3.02) |  | 1.02 (0.50-2.10) | 1.06 (0.55-2.03) | 1.12 (0.58-2.16) | 1.17 (0.49-2.82) | 1.24 (0.67-2.28) | 1.35 (0.70-2.62) | 1.58 (0.63-3.99) | 1.77 (0.92-3.40) | 1.94 (1.19-3.16)* | 2.94 (1.89-4.60)* |
| 0.590 | 5 | Ruxolitinib, 0.75%, BID | 0.21 (0.02-1.96) | 0.65 (0.50-0.85)* | 0.36 (0.04-3.05) | 0.98 (0.48-2.02) |  | 1.03 (0.49-2.17) | 1.09 (0.52-2.31) | 1.15 (0.45-2.96) | 1.21 (0.60-2.45) | 1.33 (0.63-2.80) | 1.55 (0.58-4.17) | 1.74 (0.83-3.64) | 1.90 (1.04-3.47)* | 2.88 (1.63-5.09)* |
| 0.589 | 6 | Difamilast, 1%, BID | 0.21 (0.02-1.85) | 0.63 (0.31-1.28) | 0.35 (0.04-2.88) | 0.95 (0.49-1.82) | 0.97 (0.46-2.03) |  | 1.06 (0.78-1.43) | 1.11 (0.45-2.72) | 1.17 (0.62-2.21) | 1.28 (0.65-2.54) | 1.50 (0.59-3.84) | 1.68 (0.86-3.30) | 1.84 (1.09-3.09)* | 2.79 (1.73-4.50)* |
| 0.536 | 7 | Difamilast, 0.3%, BID | 0.19 (0.02-1.76) | 0.60 (0.29-1.22) | 0.33 (0.04-2.73) | 0.90 (0.46-1.73) | 0.91 (0.43-1.93) | 0.95 (0.70-1.27) |  | 1.05 (0.43-2.58) | 1.11 (0.58-2.10) | 1.21 (0.61-2.41) | 1.42 (0.55-3.64) | 1.59 (0.81-3.13) | 1.74 (1.03-2.94)* | 2.64 (1.62-4.28)* |
| 0.513 | 8 | Tapinarof, 1%, QD | 0.19 (0.02-1.80) | 0.57 (0.23-1.43) | 0.31 (0.03-2.81) | 0.85 (0.35-2.05) | 0.87 (0.34-2.24) | 0.90 (0.37-2.20) | 0.95 (0.39-2.34) |  | 1.05 (0.44-2.50) | 1.15 (0.47-2.84) | 1.35 (0.77-2.38) | 1.51 (0.62-3.69) | 1.65 (0.76-3.62) | 2.51 (1.18-5.35)* |
| 0.466 | 9 | Roflumilast, 0.05%, QD | 0.18 (0.02-1.56) | 0.54 (0.28-1.05) | 0.30 (0.04-2.43) | 0.81 (0.44-1.49) | 0.83 (0.41-1.67) | 0.85 (0.45-1.61) | 0.90 (0.48-1.71) | 0.95 (0.40-2.25) |  | 1.09 (0.58-2.08) | 1.28 (0.52-3.18) | 1.43 (0.76-2.70) | 1.57 (0.99-2.50) | 2.38 (1.57-3.62)* |
| 0.402 | 10 | Tapinarof, 1%, BID | 0.16 (0.02-1.45) | 0.49 (0.24-1.01) | 0.27 (0.03-2.25) | 0.74 (0.38-1.43) | 0.75 (0.36-1.59) | 0.78 (0.39-1.54) | 0.82 (0.41-1.64) | 0.87 (0.35-2.13) | 0.91 (0.48-1.74) |  | 1.17 (0.46-3.01) | 1.31 (0.66-2.58) | 1.43 (0.85-2.43) | 2.17 (1.34-3.54)* |
| 0.307 | 11 | Tapinarof, 0.5%, QD | 0.14 (0.01-1.36) | 0.42 (0.16-1.10) | 0.23 (0.03-2.12) | 0.63 (0.25-1.59) | 0.64 (0.24-1.73) | 0.67 (0.26-1.70) | 0.70 (0.27-1.81) | 0.74 (0.42-1.30) | 0.78 (0.31-1.94) | 0.85 (0.33-2.19) |  | 1.12 (0.44-2.85) | 1.22 (0.53-2.81) | 1.86 (0.83-4.16) |
| 0.228 | 12 | Pimecrolimus, 1%, BID | 0.12 (0.01-1.10) | 0.38 (0.19-0.76)* | 0.21 (0.03-1.72) | 0.56 (0.29-1.08) | 0.58 (0.27-1.21) | 0.60 (0.30-1.17) | 0.63 (0.32-1.24) | 0.66 (0.27-1.62) | 0.70 (0.37-1.31) | 0.76 (0.39-1.51) | 0.89 (0.35-2.28) |  | 1.09 (0.71-1.68) | 1.66 (1.03-2.67)* |
| 0.155 | 13 | Crisaborole, 2%, BID | 0.11 (0.01-0.97)* | 0.34 (0.20-0.60)* | 0.19 (0.02-1.50) | 0.52 (0.32-0.84)* | 0.53 (0.29-0.96)* | 0.54 (0.32-0.92)* | 0.58 (0.34-0.97)* | 0.60 (0.28-1.32) | 0.64 (0.40-1.02) | 0.70 (0.41-1.18) | 0.82 (0.36-1.88) | 0.91 (0.60-1.40) |  | 1.52 (1.24-1.86)* |
| 0.010 | 14 | Vehicle | 0.07 (0.01-0.63)* | 0.23 (0.13-0.38)* | 0.12 (0.02-0.98)* | 0.34 (0.22-0.53)* | 0.35 (0.20-0.61)* | 0.36 (0.22-0.58)* | 0.38 (0.23-0.62)* | 0.40 (0.19-0.85)* | 0.42 (0.28-0.64)* | 0.46 (0.28-0.75)* | 0.54 (0.24-1.21) | 0.60 (0.37-0.97)* | 0.66 (0.54-0.81)* |  |

Each cell presents the risk ratio and 95% confidence interval for the column treatment versus the row treatment. For IGA treatment success, a risk ratio greater than 1 favors the column treatment. P-scores were used to rank treatment strategies, with higher P-scores indicating a higher probability of greater efficacy. Rank 1 indicates the highest-ranked treatment strategy. *, statistically significant. BID, twice daily; IGA, Investigator’s Global Assessment; QD, once daily.

# Supplement Table 6. P-scores, treatment rankings, and league table for IGA treatment success in the sensitivity analysis.

| P-score | Rank | Treatment | Delgocitinib, 0.5%, BID | Ruxolitinib, 1.5%, BID | Delgocitinib, 0.25%, BID | Roflumilast, 0.15%, QD | Ruxolitinib, 0.75%, BID | Difamilast, 1%, BID | Difamilast, 0.3%, BID | Tapinarof, 1%, QD | Roflumilast, 0.05%, QD | Tapinarof, 1%, BID | Tapinarof, 0.5%, QD | Crisaborole, 2%, BID | Vehicle |
| --- | --- | --- | --- | --- | --- | --- | --- | --- | --- | --- | --- | --- | --- | --- | --- |
| 0.924 | 1 | Delgocitinib, 0.5%, BID |  | 3.06 (0.34-27.84) | 1.69 (0.59-4.84) | 4.60 (0.51-41.09) | 4.69 (0.51-43.15) | 4.85 (0.54-43.68) | 5.13 (0.57-46.26) | 5.40 (0.56-52.43) | 5.69 (0.64-50.55) | 6.22 (0.69-56.11) | 7.29 (0.74-72.07) | 8.92 (1.04-76.87)* | 13.54 (1.59-115.54)* |
| 0.828 | 2 | Ruxolitinib, 1.5%, BID | 0.33 (0.04-2.97) |  | 0.55 (0.07-4.62) | 1.50 (0.75-2.99) | 1.53 (1.17-2.00)* | 1.58 (0.78-3.23) | 1.68 (0.82-3.43) | 1.76 (0.70-4.42) | 1.86 (0.95-3.63) | 2.03 (0.99-4.16) | 2.38 (0.91-6.23) | 2.91 (1.66-5.11)* | 4.42 (2.62-7.47)* |
| 0.803 | 3 | Delgocitinib, 0.25%, BID | 0.59 (0.21-1.70) | 1.81 (0.22-15.20) |  | 2.72 (0.33-22.42) | 2.78 (0.33-23.57) | 2.87 (0.35-23.84) | 3.04 (0.37-25.25) | 3.20 (0.36-28.69) | 3.37 (0.41-27.58) | 3.69 (0.44-30.62) | 4.32 (0.47-39.47) | 5.28 (0.67-41.88) | 8.02 (1.02-62.92)* |
| 0.595 | 4 | Roflumilast, 0.15%, QD | 0.22 (0.02-1.94) | 0.67 (0.33-1.33) | 0.37 (0.04-3.02) |  | 1.02 (0.50-2.10) | 1.06 (0.55-2.03) | 1.12 (0.58-2.16) | 1.17 (0.49-2.82) | 1.24 (0.67-2.28) | 1.35 (0.70-2.62) | 1.58 (0.63-3.99) | 1.94 (1.19-3.16)* | 2.94 (1.89-4.60)* |
| 0.562 | 5 | Ruxolitinib, 0.75%, BID | 0.21 (0.02-1.96) | 0.65 (0.50-0.85)* | 0.36 (0.04-3.05) | 0.98 (0.48-2.02) |  | 1.03 (0.49-2.17) | 1.09 (0.52-2.31) | 1.15 (0.45-2.96) | 1.21 (0.60-2.45) | 1.33 (0.63-2.80) | 1.55 (0.58-4.17) | 1.90 (1.04-3.47)* | 2.88 (1.63-5.09)* |
| 0.560 | 6 | Difamilast, 1%, BID | 0.21 (0.02-1.85) | 0.63 (0.31-1.28) | 0.35 (0.04-2.88) | 0.95 (0.49-1.82) | 0.97 (0.46-2.03) |  | 1.06 (0.78-1.43) | 1.11 (0.45-2.72) | 1.17 (0.62-2.21) | 1.28 (0.65-2.54) | 1.50 (0.59-3.84) | 1.84 (1.09-3.09)* | 2.79 (1.73-4.50)* |
| 0.505 | 7 | Difamilast, 0.3%, BID | 0.19 (0.02-1.76) | 0.60 (0.29-1.22) | 0.33 (0.04-2.73) | 0.90 (0.46-1.73) | 0.91 (0.43-1.93) | 0.95 (0.70-1.27) |  | 1.05 (0.43-2.58) | 1.11 (0.58-2.10) | 1.21 (0.61-2.41) | 1.42 (0.55-3.64) | 1.74 (1.03-2.94)* | 2.64 (1.62-4.28)* |
| 0.488 | 8 | Tapinarof, 1%, QD | 0.19 (0.02-1.80) | 0.57 (0.23-1.43) | 0.31 (0.03-2.81) | 0.85 (0.35-2.05) | 0.87 (0.34-2.24) | 0.90 (0.37-2.20) | 0.95 (0.39-2.34) |  | 1.05 (0.44-2.50) | 1.15 (0.47-2.84) | 1.35 (0.77-2.38) | 1.65 (0.76-3.62) | 2.51 (1.18-5.35)* |
| 0.433 | 9 | Roflumilast, 0.05%, QD | 0.18 (0.02-1.56) | 0.54 (0.28-1.05) | 0.30 (0.04-2.43) | 0.81 (0.44-1.49) | 0.83 (0.41-1.67) | 0.85 (0.45-1.61) | 0.90 (0.48-1.71) | 0.95 (0.40-2.25) |  | 1.09 (0.58-2.08) | 1.28 (0.52-3.18) | 1.57 (0.99-2.50) | 2.38 (1.57-3.62)* |
| 0.370 | 10 | Tapinarof, 1%, BID | 0.16 (0.02-1.45) | 0.49 (0.24-1.01) | 0.27 (0.03-2.25) | 0.74 (0.38-1.43) | 0.75 (0.36-1.59) | 0.78 (0.39-1.54) | 0.82 (0.41-1.64) | 0.87 (0.35-2.13) | 0.91 (0.48-1.74) |  | 1.17 (0.46-3.01) | 1.43 (0.85-2.43) | 2.17 (1.34-3.54)* |
| 0.283 | 11 | Tapinarof, 0.5%, QD | 0.14 (0.01-1.36) | 0.42 (0.16-1.10) | 0.23 (0.03-2.12) | 0.63 (0.25-1.59) | 0.64 (0.24-1.73) | 0.67 (0.26-1.70) | 0.70 (0.27-1.81) | 0.74 (0.42-1.30) | 0.78 (0.31-1.94) | 0.85 (0.33-2.19) |  | 1.22 (0.53-2.81) | 1.86 (0.83-4.16) |
| 0.140 | 12 | Crisaborole, 2%, BID | 0.11 (0.01-0.97)* | 0.34 (0.20-0.60)* | 0.19 (0.02-1.50) | 0.52 (0.32-0.84)* | 0.53 (0.29-0.96)* | 0.54 (0.32-0.92)* | 0.58 (0.34-0.97)* | 0.60 (0.28-1.32) | 0.64 (0.40-1.02) | 0.70 (0.41-1.18) | 0.82 (0.36-1.88) |  | 1.52 (1.24-1.86)* |
| 0.009 | 13 | Vehicle | 0.07 (0.01-0.63)* | 0.23 (0.13-0.38)* | 0.12 (0.02-0.98)* | 0.34 (0.22-0.53)* | 0.35 (0.20-0.61)* | 0.36 (0.22-0.58)* | 0.38 (0.23-0.62)* | 0.40 (0.19-0.85)* | 0.42 (0.28-0.64)* | 0.46 (0.28-0.75)* | 0.54 (0.24-1.21) | 0.66 (0.54-0.81)* |  |

Each cell presents the risk ratio and 95% confidence interval for the column treatment versus the row treatment. For IGA treatment success, a risk ratio greater than 1 favors the column treatment. P-scores were used to rank treatment strategies, with higher P-scores indicating a higher probability of greater efficacy. Rank 1 indicates the highest-ranked treatment strategy. *, statistically significant. BID, twice daily; IGA, Investigator’s Global Assessment; QD, once daily.

# Supplementary Table 7. P-scores, treatment rankings, and league table for EASI-75 in the primary network meta-analysis.

| P-score | Rank | Treatment | Delgocitinib, 0.5%, BID | Delgocitinib, 0.25%, BID | Tapinarof, 0.5%, QD | Tapinarof, 1%, QD | Difamilast, 1%, BID | Ruxolitinib, 1.5%, BID | Ruxolitinib, 0.75%, BID | Difamilast, 0.3%, BID | Tapinarof, 1%, BID | Roflumilast, 0.05%, QD | Vehicle |
| --- | --- | --- | --- | --- | --- | --- | --- | --- | --- | --- | --- | --- | --- |
| 0.839 | 1 | Delgocitinib, 0.5%, BID |  | 1.13 (0.35-3.64) | 1.57 (0.23-10.58) | 1.72 (0.25-11.63) | 2.54 (0.41-15.69) | 3.00 (0.59-15.15) | 3.23 (0.55-19.02) | 3.37 (0.54-21.01) | 3.58 (0.59-21.95) | 4.20 (0.71-24.84) | 8.10 (2.04-32.21)* |
| 0.812 | 2 | Delgocitinib, 0.25%, BID | 0.88 (0.27-2.83) |  | 1.38 (0.24-8.13) | 1.52 (0.26-8.93) | 2.24 (0.42-11.97) | 2.65 (0.62-11.32) | 2.85 (0.56-14.44) | 2.98 (0.55-16.03) | 3.16 (0.60-16.72) | 3.71 (0.73-18.86) | 7.15 (2.19-23.29)* |
| 0.702 | 3 | Tapinarof, 0.5%, QD | 0.64 (0.09-4.31) | 0.72 (0.12-4.25) |  | 1.10 (0.36-3.33) | 1.62 (0.27-9.56) | 1.91 (0.40-9.18) | 2.06 (0.37-11.57) | 2.15 (0.36-12.81) | 2.29 (0.39-13.37) | 2.68 (0.48-15.12) | 5.17 (1.38-19.33)* |
| 0.663 | 4 | Tapinarof, 1%, QD | 0.58 (0.09-3.94) | 0.66 (0.11-3.89) | 0.91 (0.30-2.77) |  | 1.48 (0.25-8.75) | 1.75 (0.36-8.41) | 1.88 (0.33-10.60) | 1.96 (0.33-11.72) | 2.09 (0.36-12.24) | 2.45 (0.43-13.84) | 4.72 (1.26-17.71)* |
| 0.521 | 5 | Difamilast, 1%, BID | 0.39 (0.06-2.44) | 0.45 (0.08-2.38) | 0.62 (0.10-3.64) | 0.68 (0.11-4.01) |  | 1.18 (0.27-5.08) | 1.27 (0.25-6.48) | 1.33 (0.43-4.09) | 1.41 (0.27-7.50) | 1.65 (0.32-8.46) | 3.19 (0.97-10.47) |
| 0.443 | 6 | Ruxolitinib, 1.5%, BID | 0.33 (0.07-1.68) | 0.38 (0.09-1.62) | 0.52 (0.11-2.50) | 0.57 (0.12-2.75) | 0.85 (0.20-3.64) |  | 1.08 (0.38-3.05) | 1.12 (0.26-4.88) | 1.19 (0.28-5.08) | 1.40 (0.34-5.69) | 2.70 (1.16-6.29)* |
| 0.412 | 7 | Ruxolitinib, 0.75%, BID | 0.31 (0.05-1.82) | 0.35 (0.07-1.77) | 0.48 (0.09-2.72) | 0.53 (0.09-2.99) | 0.78 (0.15-3.99) | 0.93 (0.33-2.63) |  | 1.04 (0.20-5.35) | 1.11 (0.22-5.58) | 1.30 (0.27-6.28) | 2.50 (0.82-7.60) |
| 0.391 | 8 | Difamilast, 0.3%, BID | 0.30 (0.05-1.85) | 0.34 (0.06-1.81) | 0.46 (0.08-2.76) | 0.51 (0.09-3.04) | 0.75 (0.24-2.31) | 0.89 (0.20-3.86) | 0.96 (0.19-4.91) |  | 1.06 (0.20-5.69) | 1.25 (0.24-6.42) | 2.40 (0.72-7.96) |
| 0.372 | 9 | Tapinarof, 1%, BID | 0.28 (0.05-1.71) | 0.32 (0.06-1.67) | 0.44 (0.07-2.56) | 0.48 (0.08-2.81) | 0.71 (0.13-3.76) | 0.84 (0.20-3.56) | 0.90 (0.18-4.54) | 0.94 (0.18-5.04) |  | 1.17 (0.23-5.93) | 2.26 (0.70-7.31) |
| 0.306 | 10 | Roflumilast, 0.05%, QD | 0.24 (0.04-1.41) | 0.27 (0.05-1.37) | 0.37 (0.07-2.10) | 0.41 (0.07-2.31) | 0.60 (0.12-3.09) | 0.71 (0.18-2.90) | 0.77 (0.16-3.72) | 0.80 (0.16-4.14) | 0.85 (0.17-4.32) |  | 1.93 (0.63-5.90) |
| 0.040 | 11 | Vehicle | 0.12 (0.03-0.49)* | 0.14 (0.04-0.46)* | 0.19 (0.05-0.72)* | 0.21 (0.06-0.80)* | 0.31 (0.10-1.03) | 0.37 (0.16-0.86)* | 0.40 (0.13-1.21) | 0.42 (0.13-1.38) | 0.44 (0.14-1.43) | 0.52 (0.17-1.59) |  |

Each cell presents the risk ratio and 95% confidence interval for the column treatment versus the row treatment. For EASI-75, a risk ratio greater than 1 favors the column treatment. P-scores were used to rank treatment strategies, with higher P-scores indicating a higher probability of greater efficacy. Rank 1 indicates the highest-ranked treatment strategy. *, statistically significant. BID, twice daily; EASI-75, at least 75% improvement in Eczema Area and Severity Index; QD, once daily.

# Supplementary Table 8. P-scores, treatment rankings, and league table for EASI-90 in the primary network meta-analysis.

| P-score | Rank | Treatment | Difamilast, 1%, BID | Ruxolitinib, 1.5%, BID | Tapinarof, 1%, QD | Difamilast, 0.3%, BID | Tapinarof, 0.5%, QD | Ruxolitinib, 0.75%, BID | Vehicle |
| --- | --- | --- | --- | --- | --- | --- | --- | --- | --- |
| 0.786 | 1 | Difamilast, 1%, BID |  | 1.28 (0.46-3.53) | 1.33 (0.33-5.34) | 1.27 (0.85-1.89) | 1.39 (0.34-5.64) | 1.63 (0.58-4.59) | 5.70 (2.53-12.82)* |
| 0.637 | 2 | Ruxolitinib, 1.5%, BID | 0.78 (0.28-2.16) |  | 1.04 (0.29-3.75) | 0.99 (0.35-2.77) | 1.08 (0.30-3.96) | 1.28 (0.94-1.73) | 4.45 (2.42-8.19)* |
| 0.581 | 3 | Tapinarof, 1%, QD | 0.75 (0.19-3.04) | 0.97 (0.27-3.49) |  | 0.95 (0.23-3.89) | 1.05 (0.61-1.80) | 1.23 (0.34-4.53) | 4.30 (1.39-13.33)* |
| 0.566 | 4 | Difamilast, 0.3%, BID | 0.79 (0.53-1.18) | 1.01 (0.36-2.83) | 1.05 (0.26-4.26) |  | 1.10 (0.27-4.51) | 1.29 (0.45-3.69) | 4.50 (1.96-10.33)* |
| 0.541 | 5 | Tapinarof, 0.5%, QD | 0.72 (0.18-2.92) | 0.92 (0.25-3.37) | 0.95 (0.56-1.64) | 0.91 (0.22-3.74) |  | 1.18 (0.32-4.36) | 4.10 (1.31-12.86)* |
| 0.388 | 6 | Ruxolitinib, 0.75%, BID | 0.61 (0.22-1.72) | 0.78 (0.58-1.06) | 0.81 (0.22-2.98) | 0.77 (0.27-2.21) | 0.85 (0.23-3.15) |  | 3.49 (1.84-6.62)* |
| 0.002 | 7 | Vehicle | 0.18 (0.08-0.40)* | 0.22 (0.12-0.41)* | 0.23 (0.08-0.72)* | 0.22 (0.10-0.51)* | 0.24 (0.08-0.76)* | 0.29 (0.15-0.54)* |  |

Each cell presents the risk ratio and 95% confidence interval for the column treatment versus the row treatment. For EASI-90, a risk ratio greater than 1 favors the column treatment. P-scores were used to rank treatment strategies, with higher P-scores indicating a higher probability of greater efficacy. Rank 1 indicates the highest-ranked treatment strategy. *, statistically significant. BID, twice daily; EASI-90, at least 75% improvement in Eczema Area and Severity Index; QD, once daily.

# Supplementary Table 9. P-scores, treatment rankings, and league table for TEAE in the primary network meta-analysis.

| P-score | Rank | Treatment | Ruxolitinib, 0.75%, BID | Ruxolitinib, 1.5%, BID | Delgocitinib, 0.5%, BID | Difamilast, 0.3%, BID | Tapinarof, 0.5%, QD | Vehicle | Tapinarof, 1%, QD | Difamilast, 1%, BID | Delgocitinib, 0.25%, BID | Crisaborole, 2%, BID | Roflumilast, 0.05%, QD |
| --- | --- | --- | --- | --- | --- | --- | --- | --- | --- | --- | --- | --- | --- |
| 0.754 | 1 | Ruxolitinib, 0.75%, BID |  | 0.87 (0.42-1.80) | 0.79 (0.26-2.42) | 0.76 (0.29-2.00) | 0.75 (0.25-2.23) | 0.71 (0.33-1.54) | 0.70 (0.24-2.08) | 0.67 (0.26-1.77) | 0.66 (0.25-1.75) | 0.64 (0.23-1.81) | 0.52 (0.18-1.52) |
| 0.662 | 2 | Ruxolitinib, 1.5%, BID | 1.15 (0.55-2.40) | | 0.91 (0.33-2.51) | 0.87 (0.37-2.04) | 0.86 (0.32-2.30) | 0.82 (0.45-1.50) | 0.81 (0.30-2.15) | 0.78 (0.34-1.79) | 0.77 (0.33-1.78) | 0.74 (0.29-1.86) | 0.60 (0.23-1.56) |
| 0.569 | 3 | Delgocitinib, 0.5%, BID | 1.27 (0.41-3.91) | 1.10 (0.40-3.05) | | 0.96 (0.35-2.65) | 0.95 (0.31-2.94) | 0.91 (0.40-2.06) | 0.89 (0.29-2.75) | 0.86 (0.31-2.34) | 0.84 (0.37-1.95) | 0.81 (0.28-2.39) | 0.67 (0.22-2.01) |
| 0.547 | 4 | Difamilast, 0.3%, BID | 1.32 (0.50-3.51) | 1.15 (0.49-2.68) | 1.04 (0.38-2.87) | | 0.99 (0.37-2.63) | 0.94 (0.52-1.72) | 0.93 (0.35-2.46) | 0.89 (0.49-1.61) | 0.88 (0.38-2.04) | 0.85 (0.34-2.13) | 0.69 (0.27-1.79) |
| 0.527 | 5 | Tapinarof, 0.5%, QD | 1.34 (0.45-4.01) | 1.16 (0.43-3.11) | 1.05 (0.34-3.27) | 1.01 (0.38-2.71) | | 0.96 (0.44-2.08) | 0.94 (0.43-2.04) | 0.90 (0.34-2.39) | 0.89 (0.33-2.37) | 0.86 (0.30-2.45) | 0.70 (0.24-2.05) |
| 0.485 | 6 | Vehicle | 1.40 (0.65-3.03) | 1.22 (0.67-2.22) | 1.10 (0.49-2.50) | 1.06 (0.58-1.93) | 1.05 (0.48-2.28) | | 0.98 (0.45-2.12) | 0.94 (0.53-1.69) | 0.93 (0.51-1.68) | 0.90 (0.44-1.81) | 0.73 (0.35-1.53) |
| 0.472 | 7 | Tapinarof, 1%, QD | 1.43 (0.48-4.24) | 1.24 (0.47-3.29) | 1.12 (0.36-3.46) | 1.08 (0.41-2.86) | 1.06 (0.49-2.31) | 1.02 (0.47-2.20) | | 0.96 (0.36-2.52) | 0.95 (0.36-2.51) | 0.91 (0.32-2.59) | 0.75 (0.26-2.17) |
| 0.427 | 8 | Difamilast, 1%, BID | 1.49 (0.57-3.91) | 1.29 (0.56-2.98) | 1.17 (0.43-3.20) | 1.12 (0.62-2.03) | 1.11 (0.42-2.94) | 1.06 (0.59-1.90) | 1.04 (0.40-2.74) | | 0.99 (0.43-2.27) | 0.95 (0.38-2.37) | 0.78 (0.30-2.00) |
| 0.418 | 9 | Delgocitinib, 0.25%, BID | 1.51 (0.57-3.98) | 1.31 (0.56-3.04) | 1.18 (0.51-2.74) | 1.14 (0.49-2.64) | 1.12 (0.42-2.99) | 1.07 (0.59-1.94) | 1.06 (0.40-2.79) | 1.01 (0.44-2.33) | | 0.96 (0.38-2.41) | 0.79 (0.31-2.03) |
| 0.395 | 10 | Crisaborole, 2%, BID | 1.56 (0.55-4.43) | 1.36 (0.54-3.42) | 1.23 (0.42-3.62) | 1.18 (0.47-2.97) | 1.17 (0.41-3.33) | 1.12 (0.55-2.25) | 1.10 (0.39-3.11) | 1.05 (0.42-2.62) | 1.04 (0.41-2.60) | | 0.82 (0.30-2.27) |
| 0.245 | 11 | Roflumilast, 0.05%, QD | 1.91 (0.66-5.53) | 1.65 (0.64-4.28) | 1.50 (0.50-4.51) | 1.44 (0.56-3.73) | 1.42 (0.49-4.16) | 1.36 (0.65-2.84) | 1.34 (0.46-3.88) | 1.28 (0.50-3.28) | 1.27 (0.49-3.26) | 1.22 (0.44-3.37) | |

Each cell presents the risk ratio and 95% confidence interval for the column treatment versus the row treatment. For TEAE, a risk ratio less than 1 favors the column treatment. P-scores were used to rank treatment strategies, with lower P-scores indicating a higher probability of better safety. Rank 1 indicates the highest-ranked treatment strategy. BID, twice daily; QD, once daily; TEAE, treatment-emergent adverse event.

# Supplementary Table 10. P-scores, treatment rankings, and league table for TRAE in the primary network meta-analysis.

| P-score | Rank | Treatment | Tapinarof, 0.5%, QD | Tapinarof, 1%, QD | Difamilast, 1%, BID | Difamilast, 0.3%, BID | Delgocitinib, 0.5%, BID | Vehicle | Ruxolitinib, 1.5%, BID | Roflumilast, 0.05%, QD | Ruxolitinib, 0.75%, BID | Delgocitinib, 0.25%, BID | Crisaborole, 2%, BID |
| --- | --- | --- | --- | --- | --- | --- | --- | --- | --- | --- | --- | --- | --- |
| 0.763 | 1 | Tapinarof, 0.5%, QD | | 0.88 (0.32-2.39) | 0.80 (0.22-2.91) | 0.73 (0.18-2.96) | 0.81 (0.07-9.51) | 0.60 (0.24-1.49) | 0.51 (0.10-2.56) | 0.49 (0.13-1.80) | 0.46 (0.10-2.26) | 0.39 (0.06-2.43) | 0.36 (0.13-1.03) |
| 0.702 | 2 | Tapinarof, 1%, QD | 1.14 (0.42-3.09) | | 0.90 (0.26-3.20) | 0.84 (0.21-3.26) | 0.93 (0.08-10.63) | 0.68 (0.29-1.62) | 0.59 (0.12-2.83) | 0.56 (0.16-1.98) | 0.53 (0.11-2.50) | 0.44 (0.07-2.70) | 0.41 (0.15-1.12) |
| 0.650 | 3 | Difamilast, 1%, BID | 1.26 (0.34-4.61) | 1.11 (0.31-3.91) | | 0.92 (0.30-2.80) | 1.02 (0.09-12.02) | 0.75 (0.30-1.90) | 0.65 (0.13-3.24) | 0.61 (0.17-2.28) | 0.58 (0.12-2.86) | 0.49 (0.08-3.07) | 0.46 (0.16-1.31) |
| 0.600 | 4 | Difamilast, 0.3%, BID | 1.36 (0.34-5.48) | 1.20 (0.31-4.66) | 1.08 (0.36-3.29) | | 1.11 (0.09-13.69) | 0.82 (0.29-2.34) | 0.70 (0.13-3.79) | 0.66 (0.16-2.71) | 0.63 (0.12-3.35) | 0.53 (0.08-3.56) | 0.50 (0.15-1.59) |
| 0.598 | 5 | Delgocitinib, 0.5%, BID | 1.23 (0.11-14.40) | 1.08 (0.09-12.42) | 0.98 (0.08-11.49) | 0.90 (0.07-11.17) | | 0.74 (0.08-7.25) | 0.63 (0.05-8.85) | 0.60 (0.05-7.08) | 0.57 (0.04-7.88) | 0.48 (0.04-5.63) | 0.45 (0.04-4.64) |
| 0.495 | 6 | Vehicle | 1.67 (0.67-4.15) | 1.46 (0.62-3.47) | 1.33 (0.53-3.34) | 1.22 (0.43-3.51) | 1.35 (0.14-13.31) | | 0.86 (0.23-3.21) | 0.81 (0.32-2.07) | 0.77 (0.21-2.82) | 0.65 (0.13-3.17) | 0.61 (0.37-1.00) |
| 0.427 | 7 | Ruxolitinib, 1.5%, BID | 1.94 (0.39-9.67) | 1.71 (0.35-8.26) | 1.55 (0.31-7.74) | 1.43 (0.26-7.72) | 1.58 (0.11-22.11) | 1.17 (0.31-4.36) | | 0.95 (0.19-4.77) | 0.90 (0.34-2.42) | 0.75 (0.10-5.96) | 0.71 (0.17-2.90) |
| 0.382 | 8 | Roflumilast, 0.05%, QD | 2.05 (0.56-7.56) | 1.80 (0.51-6.41) | 1.63 (0.44-6.06) | 1.51 (0.37-6.14) | 1.67 (0.14-19.65) | 1.23 (0.48-3.13) | 1.05 (0.21-5.30) | | 0.95 (0.19-4.68) | 0.79 (0.13-5.03) | 0.75 (0.26-2.15) |
| 0.371 | 9 | Ruxolitinib, 0.75%, BID | 2.16 (0.44-10.50) | 1.89 (0.40-8.96) | 1.71 (0.35-8.40) | 1.58 (0.30-8.39) | 1.75 (0.13-24.20) | 1.29 (0.35-4.72) | 1.11 (0.41-2.97) | 1.05 (0.21-5.18) | | 0.83 (0.11-6.50) | 0.78 (0.20-3.14) |
| 0.315 | 10 | Delgocitinib, 0.25%, BID | 2.58 (0.41-16.21) | 2.27 (0.37-13.90) | 2.05 (0.33-12.96) | 1.90 (0.28-12.81) | 2.10 (0.18-24.82) | 1.55 (0.31-7.63) | 1.33 (0.17-10.52) | 1.26 (0.20-7.99) | 1.20 (0.15-9.33) | | 0.94 (0.18-5.00) |
| 0.198 | 11 | Crisaborole, 2%, BID | 2.75 (0.97-7.77) | 2.41 (0.89-6.54) | 2.18 (0.76-6.24) | 2.02 (0.63-6.47) | 2.23 (0.22-23.15) | 1.65 (1.00-2.72) | 1.41 (0.34-5.79) | 1.34 (0.47-3.86) | 1.27 (0.32-5.10) | 1.06 (0.20-5.65) | |

Each cell presents the risk ratio and 95% confidence interval for the column treatment versus the row treatment. For TRAE, a risk ratio less than 1 favors the column treatment. P-scores were used to rank treatment strategies, with lower P-scores indicating a higher probability of better safety. Rank 1 indicates the highest-ranked treatment strategy. BID, twice daily; QD, once daily; TRAE, treatment-related adverse event.

# Supplementary Table 11. CINeMA assessment of confidence in the primary active-versus-vehicle network estimates.

| Outcome | Comparison | RR (95% CI) | Within-study bias | Reporting bias | Indirectness | Imprecision | Heterogeneity | Incoherence | Overall confidence | Main reasons for downgrading |
| --- | --- | --- | --- | --- | --- | --- | --- | --- | --- | --- |
| IGA-TS | Crisaborole, 2%, BID vs vehicle | 1.52 (1.24-1.86) | No concerns | No concerns | Some concerns | No concerns | No concerns | No concerns | Moderate | Indirectness |
| IGA-TS | Delgocitinib, 0.25%, BID vs vehicle | 8.02 (1.02-62.92) | No concerns | No concerns | No concerns | No concerns | Major concerns | No concerns | Low | Heterogeneity |
| IGA-TS | Delgocitinib, 0.5%, BID vs vehicle | 13.54 (1.59-115.54) | No concerns | No concerns | No concerns | No concerns | Major concerns | No concerns | Low | Heterogeneity |
| IGA-TS | Difamilast, 0.3%, BID vs vehicle | 2.64 (1.62-4.28) | No concerns | No concerns | No concerns | No concerns | No concerns | No concerns | High | None |
| IGA-TS | Difamilast, 1%, BID vs vehicle | 2.79 (1.73-4.50) | No concerns | No concerns | No concerns | No concerns | No concerns | No concerns | High | None |
| IGA-TS | Roflumilast, 0.05%, QD vs vehicle | 2.38 (1.57-3.62) | No concerns | No concerns | No concerns | No concerns | No concerns | No concerns | High | None |
| IGA-TS | Roflumilast, 0.15%, QD vs vehicle | 2.94 (1.89-4.60) | No concerns | No concerns | Some concerns | No concerns | No concerns | No concerns | Moderate | Indirectness |
| IGA-TS | Ruxolitinib, 0.75%, BID vs vehicle | 2.88 (1.63-5.09) | No concerns | No concerns | No concerns | No concerns | No concerns | No concerns | High | None |
| IGA-TS | Ruxolitinib, 1.5%, BID vs vehicle | 4.42 (2.62-7.47) | No concerns | No concerns | No concerns | No concerns | No concerns | No concerns | High | None |
| IGA-TS | Tapinarof, 0.5%, QD vs vehicle | 1.86 (0.83-4.16) | No concerns | No concerns | No concerns | Major concerns | No concerns | No concerns | Low | Imprecision |
| IGA-TS | Tapinarof, 1%, BID vs vehicle | 2.17 (1.34-3.54) | No concerns | No concerns | Some concerns | No concerns | No concerns | No concerns | Moderate | Indirectness |
| IGA-TS | Tapinarof, 1%, QD vs vehicle | 2.51 (1.18-5.35) | No concerns | No concerns | No concerns | No concerns | Major concerns | No concerns | Low | Heterogeneity |
| EASI-75 | Delgocitinib, 0.25%, BID vs vehicle | 7.15 (2.19-23.29) | No concerns | No concerns | No concerns | No concerns | Major concerns | Some concerns | Very low | Heterogeneity, incoherence |
| EASI-75 | Delgocitinib, 0.5%, BID vs vehicle | 8.10 (2.04-32.21) | No concerns | No concerns | No concerns | No concerns | Major concerns | No concerns | Low | Heterogeneity |
| EASI-75 | Difamilast, 0.3%, BID vs vehicle | 2.40 (0.72-7.96) | No concerns | No concerns | No concerns | Major concerns | No concerns | Some concerns | Very low | Imprecision, incoherence |
| EASI-75 | Difamilast, 1%, BID vs vehicle | 3.19 (0.97-10.47) | No concerns | No concerns | No concerns | Major concerns | No concerns | Some concerns | Very low | Imprecision, incoherence |
| EASI-75 | Roflumilast, 0.05%, QD vs vehicle | 1.93 (0.63-5.90) | No concerns | No concerns | No concerns | Major concerns | No concerns | Some concerns | Very low | Imprecision, incoherence |
| EASI-75 | Ruxolitinib, 0.75%, BID vs vehicle | 2.50 (0.82-7.60) | No concerns | No concerns | No concerns | Major concerns | No concerns | No concerns | Low | Imprecision |
| EASI-75 | Ruxolitinib, 1.5%, BID vs vehicle | 2.70 (1.16-6.29) | No concerns | No concerns | No concerns | No concerns | Major concerns | Some concerns | Very low | Heterogeneity, incoherence |
| EASI-75 | Tapinarof, 0.5%, QD vs vehicle | 5.17 (1.38-19.33) | No concerns | No concerns | No concerns | No concerns | Major concerns | Some concerns | Very low | Heterogeneity, incoherence |
| EASI-75 | Tapinarof, 1%, BID vs vehicle | 2.26 (0.70-7.31) | No concerns | No concerns | Some concerns | Major concerns | No concerns | Some concerns | Very low | Indirectness, imprecision, incoherence |
| EASI-75 | Tapinarof, 1%, QD vs vehicle | 4.72 (1.26-17.71) | No concerns | No concerns | No concerns | No concerns | Major concerns | Some concerns | Very low | Heterogeneity, incoherence |
| EASI-90 | Difamilast, 0.3%, BID vs vehicle | 4.50 (1.96-10.33) | No concerns | No concerns | No concerns | No concerns | Some concerns | No concerns | Moderate | Heterogeneity |
| EASI-90 | Difamilast, 1%, BID vs vehicle | 5.70 (2.53-12.82) | No concerns | No concerns | No concerns | No concerns | Some concerns | No concerns | Moderate | Heterogeneity |
| EASI-90 | Ruxolitinib, 0.75%, BID vs vehicle | 3.49 (1.84-6.62) | No concerns | No concerns | No concerns | No concerns | Some concerns | No concerns | Moderate | Heterogeneity |
| EASI-90 | Ruxolitinib, 1.5%, BID vs vehicle | 4.45 (2.42-8.19) | No concerns | No concerns | No concerns | No concerns | Some concerns | No concerns | Moderate | Heterogeneity |
| EASI-90 | Tapinarof, 0.5%, QD vs vehicle | 4.10 (1.31-12.86) | No concerns | No concerns | No concerns | No concerns | Some concerns | No concerns | Moderate | Heterogeneity |
| EASI-90 | Tapinarof, 1%, QD vs vehicle | 4.30 (1.39-13.33) | No concerns | No concerns | No concerns | No concerns | Some concerns | No concerns | Moderate | Heterogeneity |
| TEAE | Crisaborole, 2%, BID vs vehicle | 1.12 (0.55-2.25) | No concerns | No concerns | Some concerns | Major concerns | No concerns | Some concerns | Very low | Indirectness, imprecision, incoherence |
| TEAE | Delgocitinib, 0.25%, BID vs vehicle | 1.07 (0.59-1.94) | No concerns | No concerns | No concerns | Major concerns | No concerns | Some concerns | Very low | Imprecision, incoherence |
| TEAE | Delgocitinib, 0.5%, BID vs vehicle | 0.91 (0.40-2.06) | No concerns | No concerns | No concerns | Major concerns | No concerns | No concerns | Low | Imprecision |
| TEAE | Difamilast, 0.3%, BID vs vehicle | 0.94 (0.52-1.72) | No concerns | No concerns | No concerns | Major concerns | No concerns | Some concerns | Very low | Imprecision, incoherence |
| TEAE | Difamilast, 1%, BID vs vehicle | 1.06 (0.59-1.90) | No concerns | No concerns | No concerns | Major concerns | No concerns | Some concerns | Very low | Imprecision, incoherence |
| TEAE | Roflumilast, 0.05%, QD vs vehicle | 1.36 (0.65-2.84) | No concerns | No concerns | No concerns | Major concerns | No concerns | Some concerns | Very low | Imprecision, incoherence |
| TEAE | Ruxolitinib, 0.75%, BID vs vehicle | 0.71 (0.33-1.54) | No concerns | No concerns | No concerns | Major concerns | No concerns | Some concerns | Very low | Imprecision, incoherence |
| TEAE | Ruxolitinib, 1.5%, BID vs vehicle | 0.82 (0.45-1.50) | No concerns | No concerns | No concerns | Major concerns | No concerns | Some concerns | Very low | Imprecision, incoherence |
| TEAE | Tapinarof, 0.5%, QD vs vehicle | 0.96 (0.44-2.08) | No concerns | No concerns | No concerns | Major concerns | No concerns | Some concerns | Very low | Imprecision, incoherence |
| TEAE | Tapinarof, 1%, QD vs vehicle | 1.02 (0.47-2.20) | No concerns | No concerns | No concerns | Major concerns | No concerns | Some concerns | Very low | Imprecision, incoherence |
| TRAE | Crisaborole, 2%, BID vs vehicle | 1.65 (1.00-2.72) | No concerns | No concerns | Some concerns | Major concerns | No concerns | No concerns | Very low | Indirectness, imprecision |
| TRAE | Delgocitinib, 0.25%, BID vs vehicle | 1.55 (0.31-7.63) | No concerns | No concerns | No concerns | Major concerns | No concerns | No concerns | Low | Imprecision |
| TRAE | Delgocitinib, 0.5%, BID vs vehicle | 0.74 (0.08-7.25) | No concerns | No concerns | No concerns | Major concerns | No concerns | No concerns | Low | Imprecision |
| TRAE | Difamilast, 0.3%, BID vs vehicle | 0.82 (0.29-2.34) | No concerns | No concerns | No concerns | Major concerns | No concerns | No concerns | Low | Imprecision |
| TRAE | Difamilast, 1%, BID vs vehicle | 0.75 (0.30-1.90) | No concerns | No concerns | No concerns | Major concerns | No concerns | No concerns | Low | Imprecision |
| TRAE | Roflumilast, 0.05%, QD vs vehicle | 1.23 (0.48-3.13) | No concerns | No concerns | No concerns | Major concerns | No concerns | No concerns | Low | Imprecision |
| TRAE | Ruxolitinib, 0.75%, BID vs vehicle | 1.29 (0.35-4.72) | No concerns | No concerns | No concerns | Major concerns | No concerns | No concerns | Low | Imprecision |
| TRAE | Ruxolitinib, 1.5%, BID vs vehicle | 1.17 (0.31-4.36) | No concerns | No concerns | No concerns | Major concerns | No concerns | No concerns | Low | Imprecision |
| TRAE | Tapinarof, 0.5%, QD vs vehicle | 0.60 (0.24-1.49) | No concerns | No concerns | No concerns | Major concerns | No concerns | No concerns | Low | Imprecision |
| TRAE | Tapinarof, 1%, QD vs vehicle | 0.68 (0.29-1.62) | No concerns | No concerns | No concerns | Major concerns | No concerns | No concerns | Low | Imprecision |

BID, twice daily; CINeMA, Confidence in Network Meta-Analysis; EASI-75, at least 75% improvement in Eczema Area and Severity Index; EASI-90, at least 90% improvement in Eczema Area and Severity Index; IGA-TS, Investigator’s Global Assessment treatment success; QD, once daily; RR, risk ratio; TEAE, treatment-emergent adverse event; TRAE, treatment-related adverse event.

# Supplementary Figure 1. Traffic light plot of study-level risk-of-bias assessment using the Cochrane RoB 2 tool.

Risk of bias was assessed using the revised Cochrane risk-of-bias tool for randomized trials (RoB 2). Judgments were made across five domains: bias arising from the randomization process, bias due to deviations from intended interventions, bias due to missing outcome data, bias in measurement of the outcome, and bias in selection of the reported result. The overall judgment for each study was determined based on domain-level assessments. Green indicates low risk of bias, yellow indicates some concerns, and red indicates high risk of bias.

# Supplementary Figure 2. Sensitivity analysis of the primary network meta-analysis for IGA treatment success.

(A) Network plot and (B) forest plot for IGA treatment success after excluding the phase 4 open-label active-controlled trial. In the network plot, each node represents an eligible treatment strategy, node size is proportional to the number of randomized pediatric participants, and line thickness is proportional to the number of randomized controlled trials directly comparing the connected treatment strategies. Clinical trial identifiers are displayed adjacent to each treatment strategy. In the forest plot, risk ratios and 95% confidence intervals are shown for each treatment strategy compared with vehicle. Green indicates PDE4 inhibitors, dark blue indicates JAK inhibitors, and cyan-blue indicates AhR agonists. AhR, aryl hydrocarbon receptor; BID, twice daily; Cri, crisaborole; Del, delgocitinib; Dif, difamilast; IGA, Investigator’s Global Assessment; IGA-TS, Investigator’s Global Assessment treatment success; JAK, Janus kinase; PDE4, phosphodiesterase-4; QD, once daily; Rof, roflumilast; Rux, ruxolitinib; Tap, tapinarof.

# Supplementary Figure 3. Network plots of the timepoint subgroup analyses.

Network plots are shown for the 4-week subgroup, including (A) IGA-TS, (B) EASI-75, and (C) TEAE and TRAE, and for the 8-week subgroup, including (D) IGA-TS and EASI-75, (E) EASI-90 and TEAE, and (F) TRAE. Each node represents an eligible treatment strategy, with node size proportional to the number of randomized pediatric participants contributing to the corresponding network. The thickness of each line is proportional to the number of randomized controlled trials directly comparing the connected treatment strategies. Clinical trial identifiers are displayed adjacent to each treatment strategy. Green indicates PDE4 inhibitors, dark blue indicates JAK inhibitors, and cyan-blue indicates AhR agonists. AhR, aryl hydrocarbon receptor; BID, twice daily; Cri, crisaborole; Del, delgocitinib; Dif, difamilast; EASI-75, at least 75% improvement in Eczema Area and Severity Index; EASI-90, at least 90% improvement in Eczema Area and Severity Index; IGA-TS, Investigator’s Global Assessment treatment success; JAK, Janus kinase; PDE4, phosphodiesterase-4; Pim, pimecrolimus; QD, once daily; Rof, roflumilast; Rux, ruxolitinib; Tap, tapinarof; TEAE, treatment-emergent adverse event; TRAE, treatment-related adverse event.

# Supplementary Figure 4. Supplemental week-4 efficacy analysis including trials with reported week-4 outcomes.

Network plots are shown for (A) IGA-TS, (B) EASI-75, and (C) EASI-90, forest plots are shown for (D) IGA-TS, (E) EASI-75, and (F) EASI-90. This supplemental analysis included trials with a 4-week treatment duration and trials with an 8-week treatment duration that reported efficacy outcomes at week 4. In the network plots, each node represents an eligible treatment strategy, node size is proportional to the number of randomized pediatric participants, and line thickness is proportional to the number of randomized controlled trials directly comparing the connected treatment strategies. Clinical trial identifiers are displayed adjacent to each treatment strategy. Green indicates PDE4 inhibitors, dark blue indicates JAK inhibitors, and cyan-blue indicates AhR agonists. In the forest plots, risk ratios and 95% confidence intervals are shown for each treatment strategy compared with vehicle. Risk ratios greater than 1 favor the active treatment strategy. AhR, aryl hydrocarbon receptor; BID, twice daily; Cri, crisaborole; Del, delgocitinib; Dif, difamilast; EASI-75, at least 75% improvement in Eczema Area and Severity Index; EASI-90, at least 90% improvement in Eczema Area and Severity Index; IGA-TS, Investigator’s Global Assessment treatment success; JAK, Janus kinase; PDE4, phosphodiesterase-4; Pim, pimecrolimus; QD, once daily; Rof, roflumilast; Rux, ruxolitinib; Tap, tapinarof.

# Supplementary Figure 5. Network plots for the age subgroup analyses.

Network plots are shown for participants aged <12 years, including (A) IGA-TS, (B) EASI-75, (C) EASI-90, and (D) TEAE and TRAE, and for participants aged ≥12 years, including (E) IGA-TS and (F) EASI-75, EASI-90, and TEAE. Each node represents an eligible treatment strategy, with node size proportional to the number of randomized pediatric participants contributing to the corresponding network. The thickness of each line is proportional to the number of randomized controlled trials directly comparing the connected treatment strategies. Clinical trial identifiers are displayed adjacent to each treatment strategy. Green indicates PDE4 inhibitors, dark blue indicates JAK inhibitors, and cyan-blue indicates AhR agonists. AhR, aryl hydrocarbon receptor; BID, twice daily; Cri, crisaborole; Del, delgocitinib; Dif, difamilast; EASI-75, at least 75% improvement in Eczema Area and Severity Index; EASI-90, at least 90% improvement in Eczema Area and Severity Index; IGA-TS, Investigator’s Global Assessment treatment success; JAK, Janus kinase; PDE4, phosphodiesterase-4; Pim, pimecrolimus; QD, once daily; Rof, roflumilast; Rux, ruxolitinib; Tap, tapinarof; TEAE, treatment-emergent adverse event; TRAE, treatment-related adverse event.
